# Immuno-proteomic profiling reveals aberrant immune cell regulation in the airways of individuals with ongoing post-COVID-19 respiratory disease

**DOI:** 10.1016/j.immuni.2022.01.017

**Published:** 2022-03-08

**Authors:** Bavithra Vijayakumar, Karim Boustani, Patricia P. Ogger, Artemis Papadaki, James Tonkin, Christopher M. Orton, Poonam Ghai, Kornelija Suveizdyte, Richard J. Hewitt, Sujal R. Desai, Anand Devaraj, Robert J. Snelgrove, Philip L. Molyneaux, Justin L. Garner, James E. Peters, Pallav L. Shah, Clare M. Lloyd, James A. Harker

**Affiliations:** 1 National Heart and Lung Institute, Imperial College London, London, UK; 2 Chelsea and Westminster Hospital, London, UK; 3 Royal Brompton and Harefield Hospitals, Guy’s and St Thomas’ NHS Foundation Trust, London, UK; 4 Asthma UK Centre for Allergic Mechanisms of Asthma, London, London, UK; 5 Centre for Inflammatory Disease, Department of Immunology and Inflammation, Imperial College London, London, UK; 6 Margaret Turner-Warwick Centre for Fibrosing Lung Diseases, London, UK

**Keywords:** respiratory viral infection, tissue-resident memory, COVID-19, SARS-CoV-2, airways, respiratory tract, proteomics, T cells, long COVID

## Abstract

Some patients hospitalized with acute COVID-19 suffer respiratory symptoms that persist for many months. We delineated the immune-proteomic landscape in the airways and peripheral blood of healthy controls and post-COVID-19 patients 3 to 6 months after hospital discharge. Post-COVID-19 patients showed abnormal airway (but not plasma) proteomes, with an elevated concentration of proteins associated with apoptosis, tissue repair, and epithelial injury versus healthy individuals. Increased numbers of cytotoxic lymphocytes were observed in individuals with greater airway dysfunction, while increased B cell numbers and altered monocyte subsets were associated with more widespread lung abnormalities. A one-year follow-up of some post-COVID-19 patients indicated that these abnormalities resolved over time. In summary, COVID-19 causes a prolonged change to the airway immune landscape in those with persistent lung disease, with evidence of cell death and tissue repair linked to the ongoing activation of cytotoxic T cells.

## Highlights

• Post-COVID-19 airways, but not blood, show immune and proteomic changes
• Different post-COVID-19 lung abnormalities relate to distinct immunological features
• Increased BAL cytotoxic T cells are linked to epithelial damage and airway disease
• BAL myeloid and B cell numbers correlate with the degree of lung CT abnormality

## Graphical abstract

**Figure:**
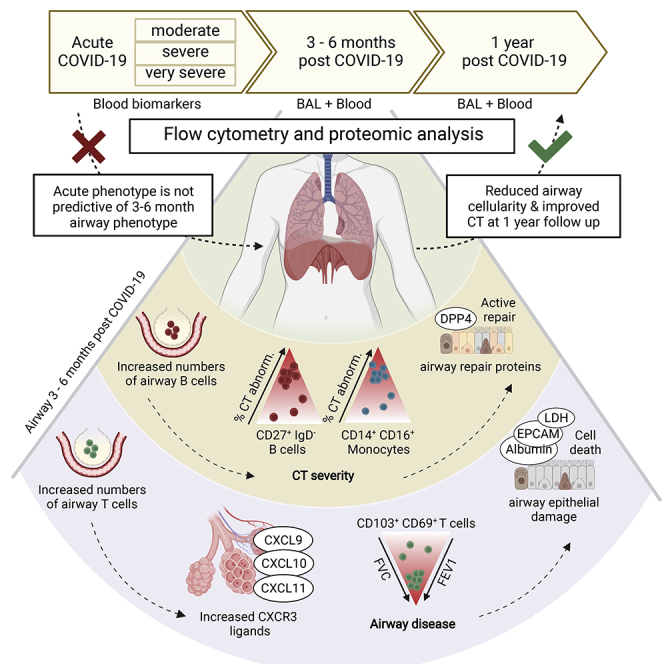

## Teaser

Many individuals recovering from acute SARS-CoV-2 infection suffer prolonged respiratory dysfunction for months to years after viral clearance. Vijayakumar, Boustani, Ogger, Papadaki et al. Show that individuals with persistent symptoms 3–6 months after infection have an altered airway immune cell landscape and evidence of ongoing lung damage. Importantly, different immune cell types correlate with the severity of distinct aspects of ongoing respiratory disease.

## Introduction

Severe acute respiratory syndrome coronavirus 2 (SARS-CoV-2)-related coronavirus disease 2019 (COVID-19) manifests as a spectrum of acute illnesses ranging from mild respiratory symptoms to severe, sometimes fatal, respiratory failure (Docherty et al., 2020). While the acute impact of COVID-19 on morbidity and mortality is well documented, we are still in the infancy of understanding the longer-term consequences. Morbidity from a range of persistent symptoms, including breathlessness, fatigue, and memory impairment, has been noted in patients recovering after the acute illness and described under the umbrella term of “long COVID” (Nalbandian et al., 2021; Sigfrid et al., 2021). Complex respiratory complications have been found in up to 18.4% of inpatients (Drake et al., 2021), and persistent breathlessness reported in more than 50% of patients recovering from COVID-19 (Mandal et al., 2021). The underlying etiology for persistent respiratory morbidity is likely to be multifactorial but may be due to persistent parenchymal abnormalities and resultant ineffective gaseous exchange. Persistent radiological abnormalities post-COVID-19 are common and may be present even up to 6 months post hospital discharge (Fabbri et al., 2021; Guler et al., 2021; Han et al., 2021; Myall et al., 2021). There is, therefore, a pressing need to understand the molecular and cellular basis of post-COVID-19 pulmonary syndromes.

The acute immunological and inflammatory events that occur during human respiratory virus infections, including SARS-CoV-2, are relatively well described (Harker and Lloyd, 2021). By contrast, the immunological landscape of the human respiratory tract after recovery from acute viral infection is poorly understood. SARS-CoV-2 infection results in the formation of long-lasting systemic immunological memory, with virus-specific antibodies and T cell responses still detectable in the majority of those infected at least 8 months post-infection and higher titers seen in previously hospitalized individuals (Dan et al., 2021). Circulating lymphocyte counts and the function and frequency of monocytes are also reduced during acute disease, although they appear to return to normal shortly after the resolution of acute disease (Mann et al., 2020; Scott et al., 2020). Likewise, plasma concentrations of inflammatory mediators such as IL-6 and CXCL10, that are highly elevated in acute disease, reduce as individuals recover (Rodriguez et al., 2020). Together, this suggests that systemic inflammatory and immune responses associated with acute disease severity resolve in line with recovery from the acute symptoms. It therefore remains unclear whether the severity of inflammation during acute disease is associated with the persistent respiratory pathology seen in some SARS-CoV-2-infected individuals months after infection or whether there is ongoing inflammation in these individuals.

This study examines the relationship between the immune system and respiratory pathology post-COVID-19. The immune cell and proteomic composition of the airways and peripheral blood were analyzed in a group of previously hospitalized COVID-19 patients with persistent radiological abnormalities in their lungs more than 3 months post discharge. In comparison to healthy individuals, the post-COVID-19 airways showed substantial increases in activated CD8^+^ and CD4^+^ tissue-resident memory (Trm) cells, and an altered monocyte pool. The airway proteome was also distinct from that observed in healthy individuals, with elevation in proteins associated with ongoing cell death, loss of barrier integrity and immune cell recruitment. None of these airway abnormalities were reflected in the proteome or immune cells of the matched peripheral blood. The scale of these alterations was not linked to the initial severity of disease while in hospital and were heterogenous; some individuals displayed heightened T cell responses associated with significant increases in CXCR3 chemokines in the airways, linked to prolonged epithelial damage and extracellular matrix (ECM) dysregulation, while other individuals exhibited a return to relative airway homeostasis. Subsequent long-term follow-up also suggested that these changes to the airway landscape progressively return to normal.

## Results

### Increased airway lymphocyte numbers characterize patients recovering from hospitalization with SARS-CoV-2

**Figure 1:**
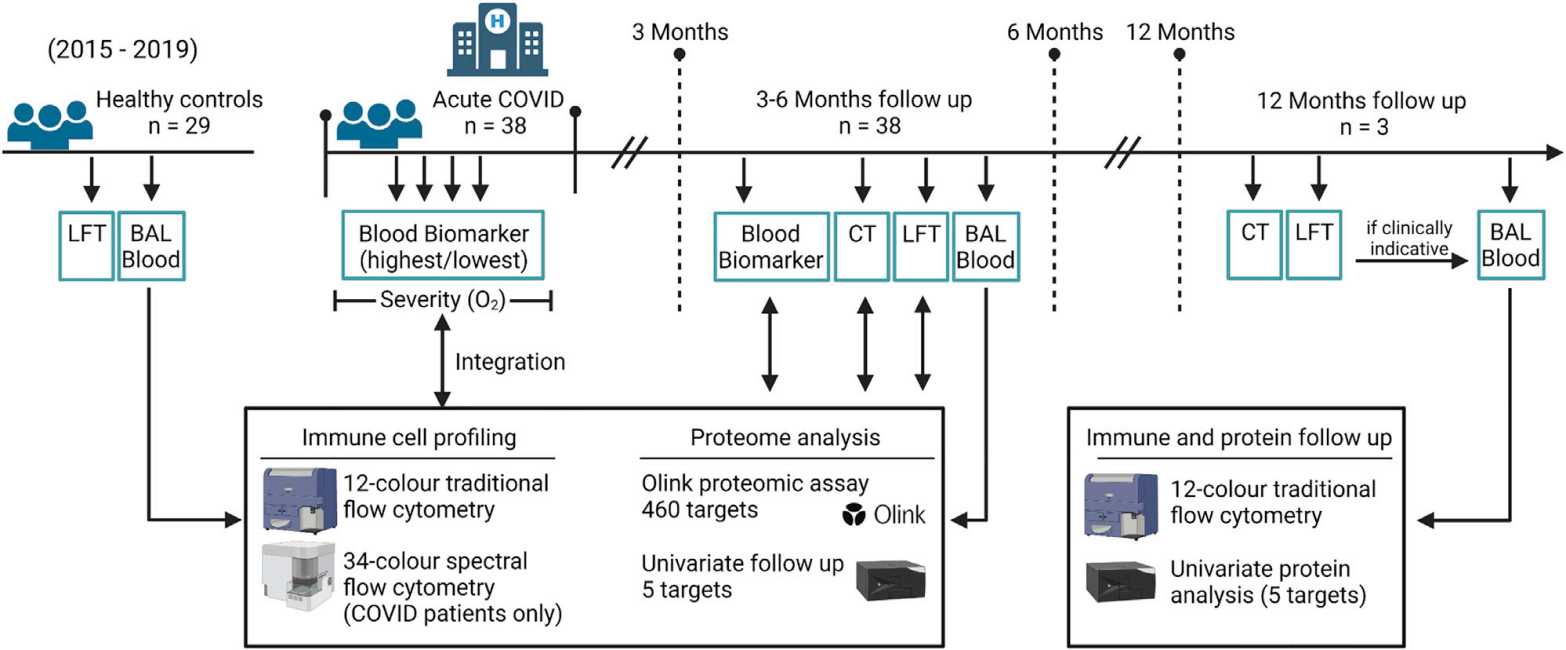
Schematic of techniques performed on airway and blood samples Schematic showing samples collected from healthy control donors (recruited 2015–2019 pre COVID-19) and from COVID-19 patients. COVID-19 patients were recruited for this study if presenting with ongoing respiratory symptoms 3 months post hospital discharge and CT and LFT were performed. Bronchoscopy was performed when clinically indicative (n = 38). Peripheral blood for subsequent analysis was obtained at time of bronchoscopy. Blood biomarker tests were performed during hospitalization and at the first follow-up visit. Immune cell profiling and proteome analysis was performed on airway (BAL) and peripheral blood (plasma) samples from healthy controls and post COVID-19 patients (3–6 months post hospitalization) using traditional and spectral flow cytometry, Olink high-throughput proteomic assay and univariate protein analysis. Immune and proteome data were integrated with acute severity and blood biomarkers during hospitalization and at first follow-up. Patients were followed-up to 12 months post-discharge. When clinically indicative a bronchoscopy was performed at this time point (n = 3). Immune cell and univariate protein analyses were performed on airway and peripheral blood (plasma) samples at this time point. LFT, lung function test; BAL, bronchoalveolar lavage; CT, computed tomography scan.

We recruited 38 patients undergoing bronchoscopy for the investigation of persistent respiratory abnormalities 3–6 months following acute SARS-CoV-2 infection (post-COVID-19) (Figure 1). All patients had ongoing respiratory symptoms and/or radiological pulmonary abnormalities on computed tomography (CT). Peripheral blood and bronchoalveolar lavage (BAL) were obtained. The post-COVID-19 cohort was stratified, based on the level of respiratory support used during their initial hospitalization with acute COVID-19, into moderate (no/minimal oxygen administered), severe (non-invasive ventilation), and very severe (invasive ventilation). We used BAL fluid, plasma, and historic flow cytometry analysis obtained from 29 healthy volunteers recruited prior to the COVID-19 pandemic as controls (demographic information in Table S1).

**Figure 2:**
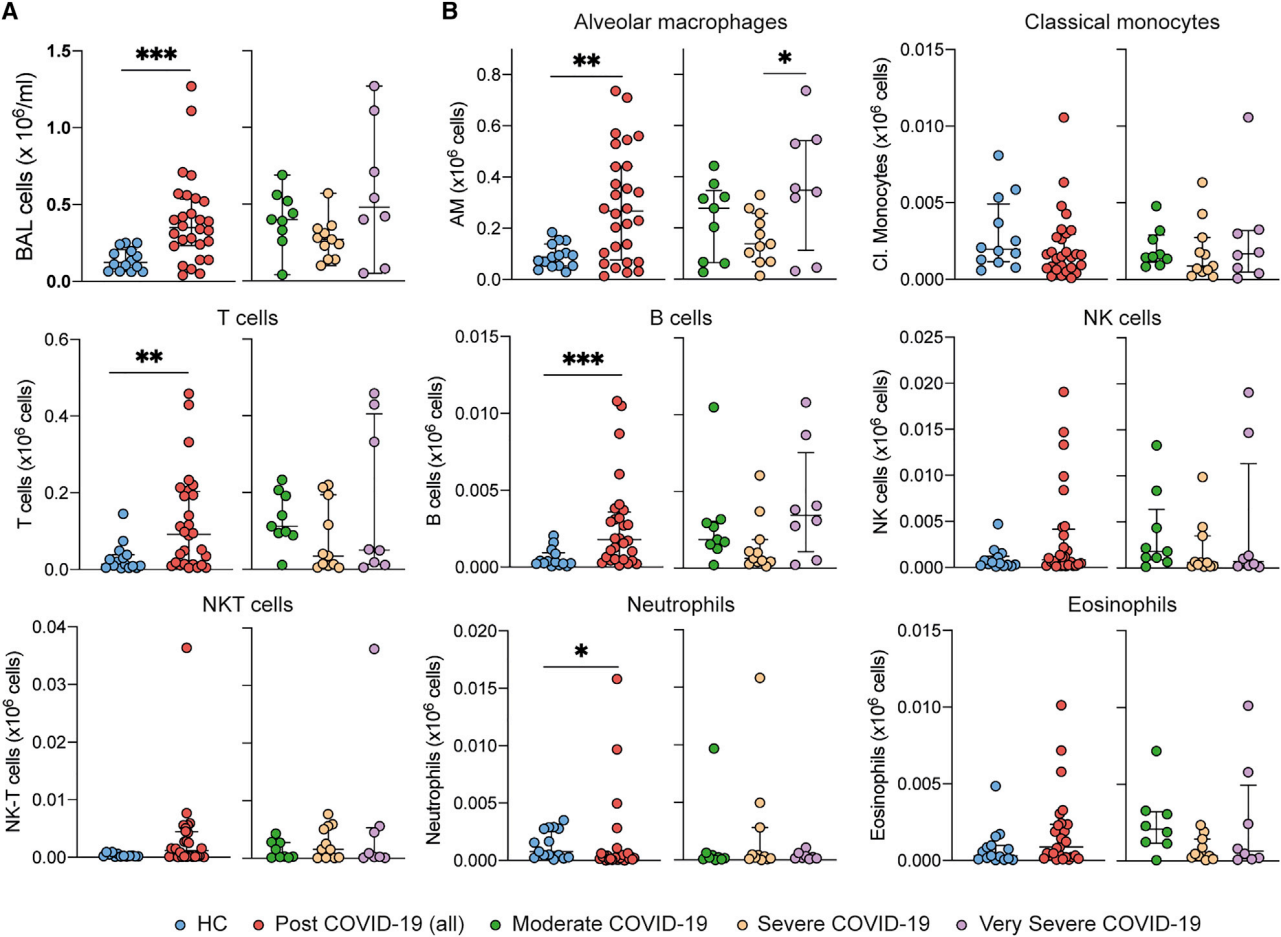
Immune cell profile is altered in post-COVID-19 BAL over 80 days after discharge (A) Left: total number of cells in BAL from healthy controls and post COVID-19 patients. Right: total number of cells in BAL from post-COVID-19 patients, stratified according to severity of the acute illness. (B) Total cell numbers of immune populations (×10^6^/mL) in BAL from healthy controls and post-COVID-19 patients, based on gating shown in STAR Methods and Figure 1. (A and B) Data are presented as mean ± SEM. Healthy controls, n = 16; post-COVID-19 patients, n = 28, moderate n = 9, severe n = 11, very severe n = 8. Statistical significance was tested by Mann-Whitney U test or one-way ANOVA + Tukey’s multiple comparison test. ^∗^p < 0.05, ^∗∗^p < 0.01, ^∗∗∗^p < 0.005. See also Figure S1.

We compared the cellular composition of BAL fluid in post-COVID-19 patients with healthy controls (HCs) by flow cytometry (Figure S1A). Post-COVID-19 patients had significantly higher numbers of cells in their airways compared with the HCs (Figure 2A). This increased cellularity was due to the elevated number of airway macrophages (AMs), T cells, and B cells (Figure 2B). Levels of CD56^+^CD3^−^ (NK, natural killer) and CD56^+^CD3^+^ (NKT) cells, CD14^+^ monocytes, and eosinophils were similar to those in HCs, while levels of neutrophils were decreased (Figure 2B). As a proportion of airway leukocytes, CD14^+^ monocytes and neutrophils were decreased in post-COVID-19 patients when compared with controls (Figure S1B).

No association between the severity of acute COVID-19 in hospital and the immune cell composition of the post-COVID-19 BAL was observed (Figure 2B). In contrast to the peripheral lymphopenia that is associated with acute COVID-19 (Chen and John Wherry, 2020), we found that in this post-COVID-19 patient cohort the frequency of T cells, B cells, and CD14^+^ monocytes in the peripheral blood was similar to HCs (Figure S1C), although the proportion of NK and NKT cells was decreased (Figure S1C). Collectively, these data indicate that after recovery from severe SARS-CoV-2 infection, immune cell frequencies in the peripheral blood are comparable to those in a group of age-matched controls. By contrast, the immune landscape of the airways remains altered, being marked by residual lymphocytes.

### Post-COVID-19 airways display a proteomic signature not reflected in blood

**Figure 3:**
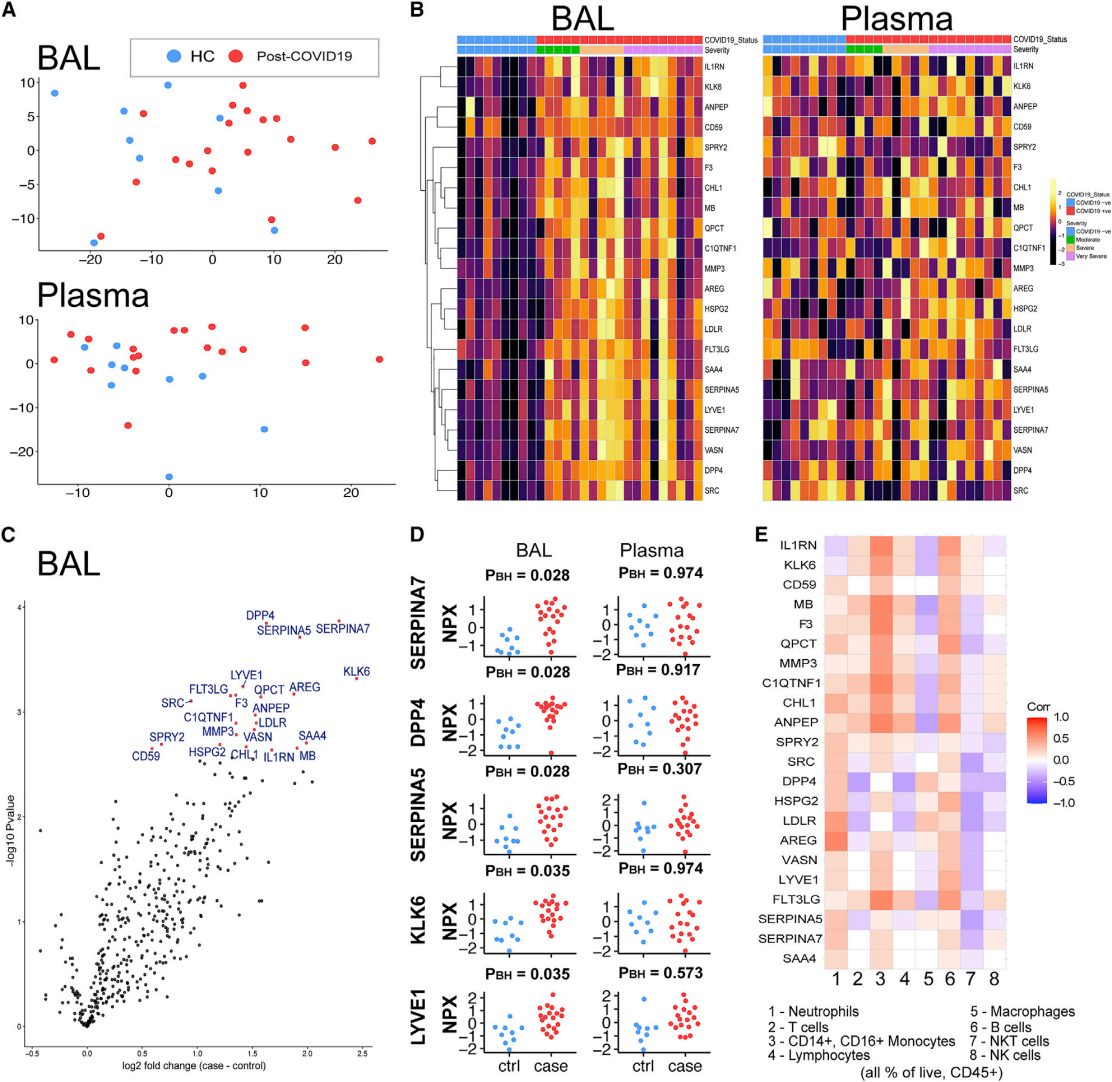
A distinct proteome is present in the post-COVID-19 airway 436 proteins in BAL and plasma were measured using Olink immunoassays in post-COVID-19 patients (n = 19) and healthy controls (n = 9). (A) Principal component analysis (PCA) of BAL and plasma proteomes: each point represents a sample. (B) Left: heatmap displaying *Z* score normalized protein abundance for the 22 proteins that were significantly differentially abundant (5% FDR) between post-COVID-19 and healthy controls in BAL. Samples have been ordered by case control status and then by peak severity during acute COVID-19 infection. Proteins are ordered by hierarchical clustering. Right: heatmap for these same 22 proteins in plasma, presented in the same order as for BAL. (C) Volcano plot showing differentially protein abundance analysis between post-COVID-19 patients and healthy controls in BAL. Nominal −log_10_ p values are shown. Significantly differentially abundant proteins (5% FDR) are colored in red and labeled. (D) BAL and plasma normalized protein abundance (NPX) expression for the 5 most significantly differentially abundance proteins between post-COVID-19 patients and healthy controls. PBH, Benjamini-Hochberg adjusted p values. (E) Correlation between the 22 differentially abundant proteins (from the analysis of post-COVID-19 versus HC) and immune cell frequency in BAL. See also Figures S2–S5.

We next evaluated the airway and blood (plasma) proteomes, using the Olink platform to measure 435 unique proteins in BAL and plasma from 19 post-COVID-19 patients and nine HCs. The proteins measured were highly enriched for immune-inflammatory processes (Tables S2A–S2C). Principal component analysis (PCA) of BAL proteomes revealed differences between post-COVID-19 patients and HCs (Figure 3A), with the separation of cases and controls most evident along PC1. In plasma, PCA also revealed differences, most evident along PC2, although the differences were less marked than for BAL. However, in both BAL and plasma there was a considerable overlap in the spatial location of post-COVID-19 and control in the PCA plots, indicating heterogeneity in post-COVID-19 patients, with some displaying similar proteomic profiles to HCs. Unsupervised hierarchical clustering revealed two major clusters in BAL, one consisting predominantly of post-COVID-19 samples and the other predominantly of HC samples (Figure S2A). By contrast, in plasma there was no visible structure to the clustering and a lack of clear separation of cases and controls (Figure S2B). These analyses indicate that the post-COVID-19 phenotype is predominantly reflected by the airway proteome rather than the peripheral blood.

Differential protein abundance analysis comparing post-COVID-19 cases with HCs identified 22 proteins in BAL with significantly altered concentrations (5% false discovery rate, FDR) (Figures 3B and 3C; Table S2D). These were all upregulated in post-COVID-19 patients compared with HCs (Figure 3C). To provide a succinct and standardized nomenclature, we report proteins by the symbols of the genes encoding them (see Table S2A for mapping to full protein names). The proteins that were most significantly differentially abundant between post-COVID-19 and controls were thyroxine-binding globulin (SERPINA7), dipeptidyl peptidase 4 (DPP4), plasma serine protease inhibitor (SERPINA5), kallikrein-related peptidase-6 (KLK6), lymphatic vessel endothelial hyaluronic acid receptor 1 (LYVE1), amphiregulin (AREG), factor 3 (F3), Fms-related tyrosine kinase 3 ligand (FLT3LG), glutaminyl-peptide cyclotransferase (QPCT), metalloproteinase-3 (MMP3), and Proto-oncogene tyrosine-protein kinase Src (SRC) (Figures 3C and 3D). Pathway annotation of the 22 upregulated proteins using STRING-db highlighted “leucocyte activation,” “regulation of cell death,” “response to injury,” and “response to wounding” (Table S2E). Analysis of the relationship between the 22 differentially abundant proteins and the airway immune cell proportions showed that neutrophils most strongly correlated with AREG and low-density lipoprotein receptor (LDLR), while monocyte proportions correlated with F3, FLT3LG, myoglobin (MB), and IL-1 receptor antagonist protein (IL1RN) (Figure 3E). Although elevated in the airways post-COVID-19, T cells displayed only weak correlations with the differentially abundant proteins.

In contrast to the BAL findings, no significant differences between protein concentrations were detected in the plasma of post-COVID-19 patients versus HCs (Table S2F). Comparison of the estimated log_2_ fold changes for the 22 proteins upregulated in post-COVID-19 BAL fluid with the estimated log_2_ fold changes for these same proteins in plasma revealed no correlation (Figures S2C and S2D), indicating that the post-COVID-19 airway proteomic signature is not reflected in the circulation.

The modest sample size and multiple testing burden of 435 proteins likely limited the statistical power to detect differentially abundant proteins. To examine whether there was evidence of signal in the proteomic data that was hidden by the hard thresholding in the differential abundance analysis, we examined quantile-quantile (QQ) plots of the distribution of expected p values under the null hypothesis of no proteomic differences between cases and controls versus the observed p values. For both BAL and plasma, the QQ plots revealed substantial deviation from the diagonal (albeit more so in BAL), indicating the presence of systematic differences between post-COVID-19 cases and HCs for plasma proteins as well as BAL proteins (Figure S3A). Corroborating this, the distribution of p values for the proteins was not uniformly distributed, with skewing toward zero (Figure S3B). This is consistent with the separation of post-COVID-19 and control samples on the PCA plots for both BAL and plasma. These data suggest that there are differences in both the BAL and plasma proteomes of post-COVID-19 cases compared with HCs, but that the effects are much stronger in BAL.

To increase power and investigate potential protein-protein relationships, we utilized a network analysis method, weighted coexpression network analysis (WGCNA) (Langfelder and Horvath, 2008; Zhang and Horvath, 2005), that leverages the correlation between proteins to enable dimension reduction, thus reducing the multiple testing burden. We used WGCNA to identify modules of correlated proteins and then tested for associations between these protein modules (represented quantitatively by an eigenprotein value) and case/control status. In BAL, this revealed two modules (“red” and “blue”) significantly associated with case/control status (5% FDR) (Tables S2G–S2I).

The red module consisted of 37 proteins (Figure S4A; Table S2H), characterized by proteins associated with chemotaxis, inflammation, cell death, and repair. In post-COVID-19 patients, we observed co-upregulation of groups of related red module proteins such as the CXCR3 chemokines (CXCL9, CXCL10, and CXCL11), and interleukin-1A (IL1A) and its antagonist IL1RN (Figures S4A and S4B). We used STRING-db to visualize known or predicted relationships between proteins in the module (Figures S4A and S4B). To highlight putative key proteins in the red and blue modules in a data-driven way, we identified hub proteins, defined as those that are highly interconnected in the proteomic network defined by WGCNA (Table S2J). This identified caspase-3 (CASP3), epithelial cell adhesion molecule (EPCAM), F3, and MB in the red module. F3 and MB, an oxygen binding protein release, which is linked to muscle damage, were also identified as upregulated in the univariate differential abundance analysis (Figures 3B and 3C). CASP3 is involved in cell death, EPCAM and keratin-19 (KRT19) are indicative of epithelial cell debris within the BAL, and transforming growth factor A (TGFA) is an EGFR ligand involved in epithelial repair. The presence of CASP3, EPCAM, KRT19, and TGFA in the red module therefore suggests that one of the key features of the post-COVID-19 airway is the presence of ongoing epithelial injury and repair.

Blue module proteins were predominantly upregulated in post-COVID-19 versus HC BAL (Figure S5A). The blue module was larger than the red module, containing 108 proteins involved in a wide range of biological activities. Several members were involved in cell adhesion and immune cell signaling. The hub proteins in the blue module were complement component C1q receptor (CD93), cartilage oligomeric matrix protein (COMP), insulin-like growth-factor-binding protein 3 (IGFBP3), interleukin-1-receptor-type 2 (IL1R2), LYVE1, MMP2 (72 kDa type IV collagenase), neural cell adhesion molecule 1 (NCAM1), L-selectin (SELL), tyrosine-protein kinase receptor Tie-1 (TIE1), Tenascin-X (TNXB), and Vasorin (VASN) (Figure S5B). Of these, LYVE1 and VASN were also identified in the differential abundance analysis.

In contrast to the BAL network analysis, no protein modules in plasma were associated with case control status. This suggests that persistent post-COVID-19 respiratory abnormalities have a demonstrable proteomic signature in BAL that is distinct from that of HCs. By contrast, we were unable to detect changes in the plasma proteome of post-COVID-19 patients, even with the enhanced statistical power provided by the WGCNA method.

There were no significant associations between the severity of initial infection and proteins in BAL fluid in the post-COVID-19 cases, paralleling our flow cytometry results. Thus, the immune-proteomic profile of the post-COVID-19 airway does not appear to relate to the severity of acute disease.

### CXCR3 ligands and markers of ongoing epithelial damage correlate with airway T Cell and monocyte responses

**Figure 4:**
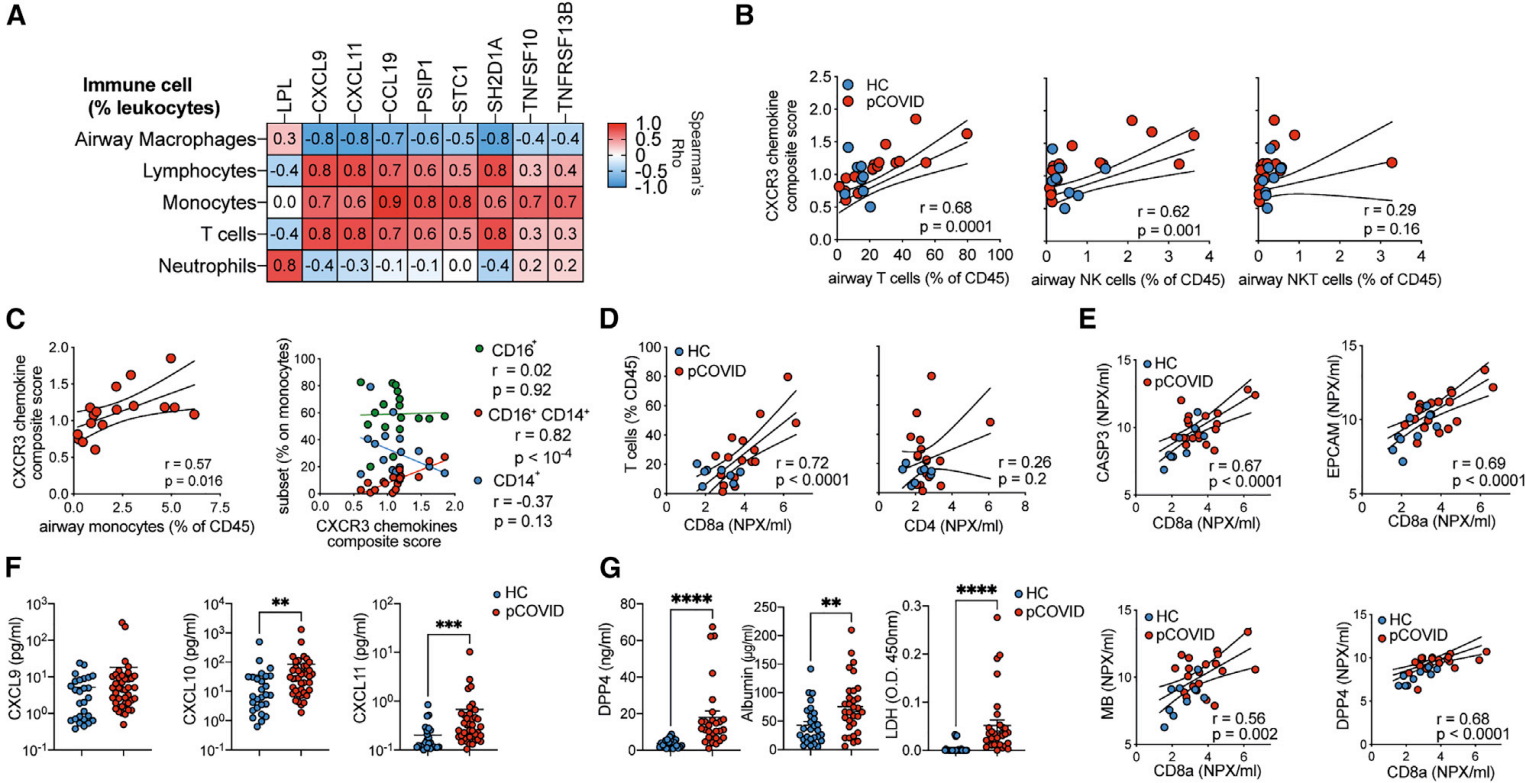
CXCR3 ligands and markers of epithelial damage correlate with CD8 T cells numbers in the airways BAL immune cells and protein concentrations were analyzed post-COVID-19 infection. (A) Heatmap displaying the relationship between proteins and immune cell frequencies. The proteins and immune cell traits displayed are those with at least one significant (5% FDR) association from linear regression analyses (see Table S2K). (B and C) For each sample, protein concentrations for CXCL9, CXCL 10, and CXCL 11 were normalized to the median concentration in healthy controls. For each sample, the mean of the normalized values for the 3 proteins was calculated to provide a summary metric for CXCR3 chemokines. This was then plotted against versus (B) T, NK, and NKT proportions in post-COVID-19 patients and healthy controls and (C) monocyte frequencies and subsets in post-COVID-19 patients only. (D) BAL T cell frequency versus CD4 and CD8a concentrations as measured by Olink. (E) CD8a concentration versus CASP3, EPCAM, MB, and DPP4 in the airways. (F) CXCL9, CXCL10, and CXCL11 concentration in in the BAL were measure by legendplex. (G) DPP4, albumin, and LDH concentrations in the BAL determined by ELISA. Data are presented as median ± IQR. (A) Pearsons correlation of n = 19 post-COVID-19 patients, the r value is shown. (B–E) Each point represents an individual patient, linear regression line ±95% confidence intervals are depicted, and r and p values from Pearsons correlation are stated. (F and G) Represents n = 38 post-COVID-19 and n = 20 healthy control individuals. Statistics were conducted using Mann-Whitney U test. ^∗^p < 0.05, ^∗∗^p < 0.01, ^∗∗∗^p < 0.005, ^∗∗∗∗^p < 0.001. pCOVID = post-COVID-19.

Given that post-COVID-19 patient airways displayed proteomic abnormalities and elevated T, B, and NK cells, we next sought to determine which BAL proteins were associated with particular immune cell populations and identified several significant associations (5% FDR) (Figure 4A; Table S2K). The proportion of monocytes in the airways was significantly associated with a range of airway proteins, including the CCR7 ligand CCL19, the CXCR3 ligands CXCL9 and CXCL11, TRAIL (TNFSF10), and BAFF (TNFSF13B) (Figure 4A). CXCL9 and CXCL11 also positively correlated with lymphocyte and T cell frequencies and negatively correlated with AM frequencies (Figure 4A). T cell frequencies positively correlated with SH2D1A (SLAM associated protein ). B, NK, and NKT cells did not significantly correlate with any protein.

In addition to displaying correlations with immune cell frequencies in the airways, the chemokines CXCL9, CXCL10, and CXCL11, and their receptor, CXCR3, are all members of the red WGCNA module that characterized the post-COVID-19 airway. Given their shared signaling pathway, we analyzed the contribution of these chemokines further by calculating a composite score (reflecting an average fold change of each chemokine relative to median concentrations found in HC BAL) and testing it for association with BAL immune cell frequencies. This CXCR3 chemokine score strongly correlated with airway T cell frequencies (r = 0.68, p = 0.0001), and with airway NK cells (r = 0.62, p = 0.001). In contrast, there was no significant correlation to airway NKT cells (p = 0.16) (Figure 4B). In the post-COVID-19 dataset (as CD16 was not present in historic flow data used for HCs), total monocyte frequencies also correlated with the CXCR3 chemokine score (r = 0.57, p = 0.016) (Figure 4C). Intermediate (CD14^+^CD16^+^) monocytes positively correlated with CXCR3 ligands, while CD14^+^ monocytes displayed a negative correlation, and CD16^+^ monocytes displayed no correlation (Figure 4C). T cell proportions in the airways correlated tightly with the concentration of CD8a protein, but not CD4, in the BAL (Figure 4D), suggesting the increased airway T cells were most likely the result of increased CD8^+^ T cell frequencies.

We next determined the relationship between T cell frequencies and other protein members of the red module, specifically those indicating ongoing epithelial damage. CD8a correlated strongly with the concentrations of CASP3 and EPCAM, concomitant with two of the differentially expressed proteins: MB and DPP4 (Figure 4E). Collectively, these data suggest that proteins linked to the recruitment of T cells, especially cytotoxic T cells, are strongly associated with proteins that are both indicative of ongoing epithelial damage and upregulated in the airways post-COVID-19.

To further evaluate this, we measured BAL CXCL9, CXCL 10, and CXCL 11 via cytometric bead immune assay in an expanded cohort of HCs (n = 29) and post-COVID-19 patients (n = 38), including those samples on which Olink data were generated plus additional samples. Analysis of this larger sample set revealed that CXCL10 and CXCL11, but not CXCL9, were significantly upregulated in post-COVID-19 BAL compared with HC BAL (Figure 4F). We also confirmed the presence of increased damage in the post-COVID-19 airway by measuring DPP4 and two markers of damage not analyzed by Olink, albumin and lactate dehydrogenase (LDH) (Figure 4G). DPP4, albumin, and LDH were significantly upregulated in the airways of patients post-COVID-19 compared with HCs, validating the observations made by Olink and confirming the presence of ongoing damage within the respiratory tracts of patients previously hospitalized for COVID-19.

### Different airway immune populations associate with distinct aspects of post-COVID-19 pathophysiology

**Figure 5:**
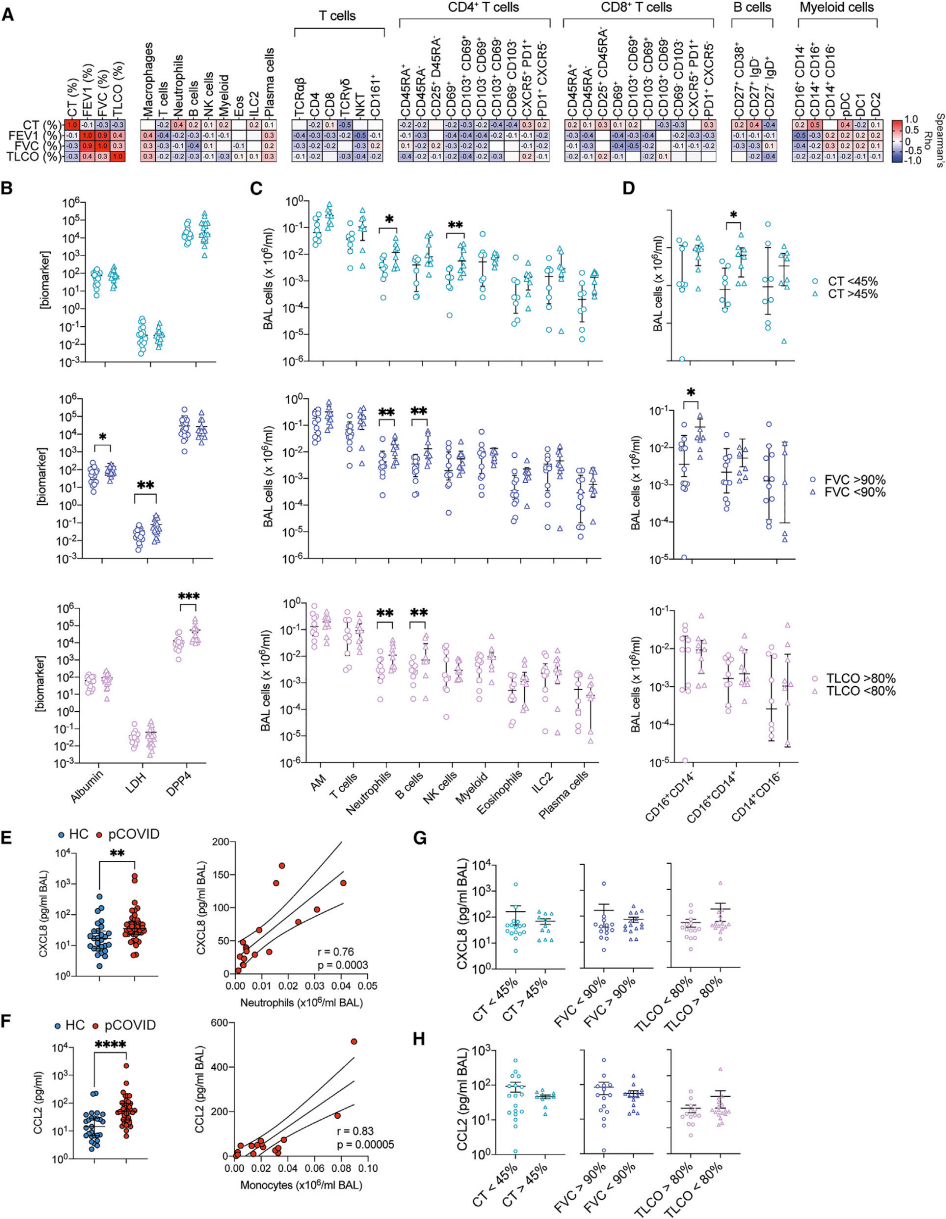
Distinct airway proteomic and immune cell phenotypes correlate with distinct indicators of respiratory pathology post-COVID (A) Immune cell proportions in the BAL, as a percentage of total leukocytes, BAL albumin (μg/mL), LDH (OD450), and DPP4 (ng/mL) were correlated with CT (% abnormality) or FEV1, FVC, and TLCO (% of predicted normal). Spearman’s rho is displayed as a heatmap. (B) Albumin (μg/mL), LDH (OD450), and DPP4 (ng/mL) in the BAL segregated by CT abnormality (%), predicted FVC (%), and predicted TLCO (%). (C) The number of major immune cell population per mL of BAL versus CT abnormality, FVC, and TLCO. (D) Total number of monocyte subsets per mL BAL was segregated by CT, FVC, and TCLO. (E) BAL CXCL8 (pg/mL) measured by Legendplex in HC and post-COVID-19 patients and correlated versus total neutrophil numbers (per mL/BAL). (F) BAL CXCL8 (pg/mL) measured by legendplex in post COVID-19 patients segregated by CT abnormality (%), predicted FVC (%), and predicted TLCO (%). (G) BAL CCL2 (pg/mL) measured by legendplex in HC and post COVID-19 patients and correlated versus myeloid cells (CD11b^+^) in the BAL. (H) BAL CCL2 (pg/mL) measured by legendplex in post COVID-19 patients segregated by CT abnormality (%), predicted FVC (%), and predicted TLCO (%). Where applicable individual points are shown, and data are presented as median ± IQR. Each point represents an individual patient. Statistical significance for (B–H) was tested by Mann-Whitney U test. Benjamini-Hochberg adjusted (5% FDR) p values ^∗^p < 0.05, ^∗∗^p < 0.01, ^∗∗∗^p < 0.005, ^∗∗∗∗^p < 0.001. Pearson’s correlations were performed in (E and G), r and p values are shown, as is a line of best fit ±95% confidence intervals. See Figures S6 and S7.

The cause of persistent respiratory symptoms post-COVID-19, and its relationship to local changes in the immune response, is critical to the understanding and treatment of post-COVID-19 respiratory disease. Therefore, we evaluated the relationship between the immune response and clinical measures of respiratory health taken shortly before bronchoscopy. Respiratory health was assessed through imaging (CT) and pulmonary function testing, including the measurement of forced expiratory volume (FEV1, the amount of air a person can force out of their lungs in 1 s), forced vital capacity (FVC, the total amount of air an individual can exhale from their lungs), and gas transfer capacity of the lungs, measured by the uptake of carbon monoxide (TLCO). There was heterogeneity within the cohort (Table S1). Pulmonary function and CT imaging were generally poorly correlated, aside from FEV1 and FVC which, given their shared relationship, tightly correlated. In particular, the degree of CT abnormality only very weakly correlated with reduced airway function (FEV1 or FVC) and gas transfer (TLCO) (Figure 5A).

To determine whether this heterogeneity in respiratory function post-COVID-19 was differentially associated with distinct immune cell phenotypes in the airways, we utilized high-parameter spectral deconvolution cytometry to analyze the expression of 33 markers on BAL immune cells. Unbiased clustering of lymphocytes and myeloid cells using FlowSOM in parallel with manual gating (Figure S6A) indicated that this approach could identify the majority of expected immune cell populations and subsets, while the absence of clusters of cells with unexpected marker expression patterns suggests the post-COVID-19 airway does not feature substantial unique immune cell types (Figures S6B and S6C). Manual gating supported this, with the enrichment of tissue-resident immune cells in the BAL, and naive lymphocyte populations in the blood (Figures S6D and S6E). The proportion of immune cells and their subsets in the BAL revealed that no one immune cell type was dominantly linked to post-COVID-19 respiratory pathophysiology (Figure 5A). Instead, different immune cell populations correlated with distinct indicators of disease. Neutrophils, CD14^+^CD16^+^ intermediate monocytes, and IgD^−^CD27^+^ memory B cells correlated most strongly with increased CT abnormality. Meanwhile, reductions in predicted FEV1 or FVC were correlated more strongly with lymphocytes, with NKT, B, and activated CD8 T cells having the strongest correlation with these measures of airway function. These correlations were not significant after a 5% FDR cutoff was applied across the multiple tests. However, similar analysis using BAL cell numbers indicated that these specific immune and clinical traits were significantly correlated with a 5% FDR cutoff (Figure S7).

Segregating the cohort based on clinical measurements supported the observation that increases in different BAL biomarkers and immune cell populations are linked to distinct clinical features. BAL DPP4, LDH, and albumin concentrations were not different in individuals with increased CT abnormality compared with those with more limited changes (Figure 5B). Albumin and LDH, but not DPP4, were increased in individuals with reduced FVC, while DPP4, but not albumin nor LDH, was increased in individuals with reduced TCLO (Figure 5B). Elevated BAL neutrophils correlated with more severe abnormalities in CT, FVC, and TLCO (Figure 5C). BAL B cells were increased in individuals with enhanced CT abnormalities, or decreased FVC, but not TCLO (Figure 5C). NK cells were increased in those with increased CT abnormalities (Figure 5C). Total myeloid cells in the airways did not associate with any specific measure of respiratory disease. However, intermediate CD16^+^CD14^+^ monocytes were increased in individuals with higher proportions of CT abnormality, while non-classical CD16^+^ monocytes were increased in individuals with reduced FVC (Figure 5D). Concomitant with enhanced BAL neutrophilia, the major neutrophil chemokine CXCL8 was increased in post-COVID-19 BAL compared with HC BAL, and CXCL8 concentration was significantly correlated with airway neutrophils (Figure 5E). Similarly, CCL2 was significantly increased in post-COVID-19 BAL, and tightly correlated to BAL monocyte numbers (Figure 5G). CXCL8 or CCL2 did not segregate with worsened CT, FVC, or TLCO (Figures 5G and 5H).

Collectively, these data highlight clinical assessments that measure distinct pathophysiological aspects of respiratory disease and are linked to different immunological components. CT abnormalities specifically were associated with granulocytic and monocytic involvement, whose presence is associated with chemokines canonical for their recruitment.

### BAL T cell and B cells display discrete relationships with ongoing respiratory disease post-COVID-19

**Figure 6:**
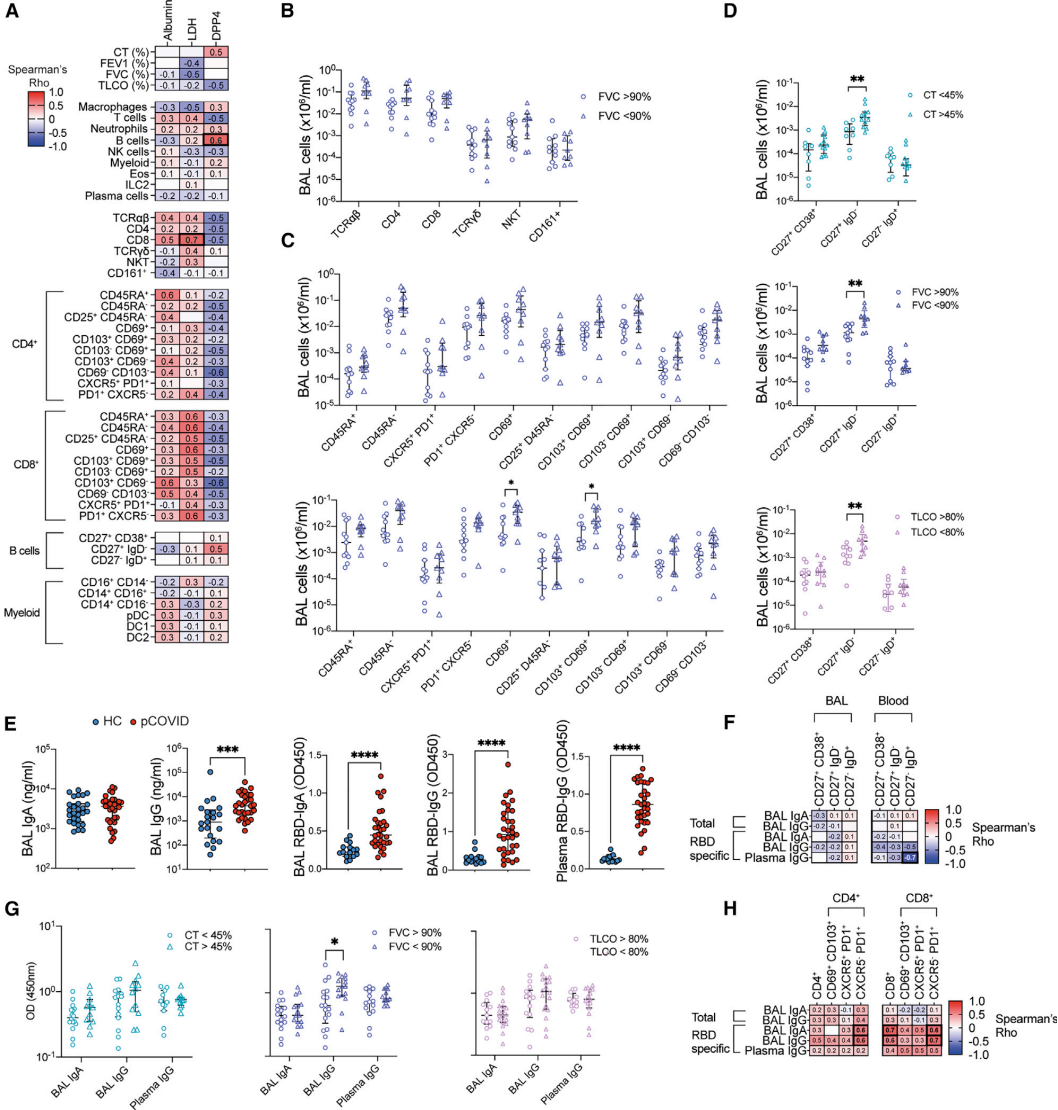
Increased airway T cell and B cell abundance is associated with more severe ongoing respiratory pathophysiology post-COVID-19 (A) Immune cell proportions in the BAL, as a percentage of total leukocytes, BAL albumin (μg/mL), LDH (OD450), and DPP4 (ng/mL) were correlated with BAL albumin, LDH, and DPP4 concentrations. (B and C) (B) BAL T cell subtypes and (C) subsets of CD4 and CD8 T cells were analyzed against FVC. (D) B cells subsets numbers per mL BAL segregated by CT, FVC, and TLCO. (E) Total and RBD-specific IgA and IgG were measured in the BAL and plasma. (F) Antibody concentrations were correlated with BAL and plasma B cell subsets of total leukocytes. (G) Antibody concentrations measured in BAL and plasma segregated by CT, FVC, and TLCO. (H) Antibody concentrations were correlated with BAL CD4 and CD8 T cells and their subsets as a proportion of total leukocytes. (A, F, and H) Spearman correlation. Correlations p < 0.05 after Benjamini-Hochberg adjustment for an FDR of 5% are indicated by thickened boxes. (B–E and G) was tested by Mann-Whitney U test. Benjamini-Hochberg adjusted (5% FDR) ^∗^p < 0.05, ^∗∗^p < 0.01, ^∗∗∗^p < 0.005, ^∗∗∗∗^p < 0.001. (A, F, and G) are display Spearman’s rho correlation.

We next carried out further correlation with the three biomarkers, DPP4 (the most differentially regulated protein in the post-COVID-19 BAL), LDH, and albumin, as markers of ongoing damage in the airways. LDH activity inversely correlated with predicted FEV1 and FVC and strongly correlated with proportions of various subsets of CD8 T cells in the BAL, with albumin showing similar, albeit weaker, links (Figure 6A). Conversely DPP4 was correlated with increased CT abnormality and reduced TCLO but negatively correlated with the proportion of T cells in the BAL. Instead, the proportion of B cells, specifically memory B cells, were the only immune cells analyzed to show a strong correlation to DPP4 concentrations (Figure 6A).

B and T cells can play a critical role both in protective and pathological immune responses during acute COVID-19 (Harker and Lloyd, 2021) and were significantly elevated in the BAL of individuals post-COVID-19 compared with HCs (Figure 2B). Correlation with clinical measurements of respiratory function and pathophysiology suggested T cells were more strongly linked to airway disease, indicated by reduced FEV1 and FVC, while B cells, specifically memory B cells, appeared to be linked to the full range of more severe pathophysiological changes seen post-COVID-19 (Figure 5A). The number of CD69^+^ CD8 T and CD103^+^CD69^+^ CD8 T cells in BAL was significantly increased among those post-COVID-19 patients with an FVC less than 90% of that predicted (Figures 6B and 6C). No other T cell population or subset showed significance in individuals with reduced FVC, but similar trends were present for activated CD4 T cells (Figures 6B and 6C). Conversely, analysis of B cells revealed that individuals with increased CT abnormality or reduced FVC or TCLO had significantly increased memory B cells in their airways, while naive B cells and plasmablasts were not different (Figure 6D).

To examine the role of B cells in ongoing respiratory dysfunction further, antibody responses were measured. While total IgA in the BAL was similar in HCs versus post-COVID-19 patients, total IgG was significantly increased (Figure 6E). As would be expected, despite samples having been taken pre-vaccination, post-COVID-19 patients also had detectable antibodies against the receptor binding domain (RBD) of SARS-CoV-2’s spike protein, with IgA and IgG abundance in the BAL, and IgG in the plasma (Figure 6E). The total or virus-specific antibody concentrations present post-COVID-19 displayed minimal correlation with the proportion of B cell subsets found either in the BAL or systemically (Figure 6F). Instead, BAL virus-specific IgG was significantly increased in individuals with reduced FVC, but not in individuals with increased CT abnormalities or reduced TLCO (Figure 6G). In line with this, virus-specific antibodies correlated tightly with CD4 and CD8 T cells in the BAL (Figure 6H). There was a particular correlation with activated (e.g., PD1^+^ CXCR5^−^ CD4 and CD8) T cells, rather than CXCR5^+^ PD1^+^ CD4 and CD8 T cells, which are more canonically associated with B cell helper functions.

Collectively, these data suggest that heightened T cell frequencies, especially CD8^+^ Trm cells, are associated with increased indicators of cell death and ongoing airway disease post-COVID-19. Meanwhile, the presence of memory B cells in the BAL was linked to increased DPP4 (but not LDH) and a range of pathophysiological outcomes post-COVID-19.

### The post-COVID-19 airway immune cell infiltrates decline over time

**Figure 7:**
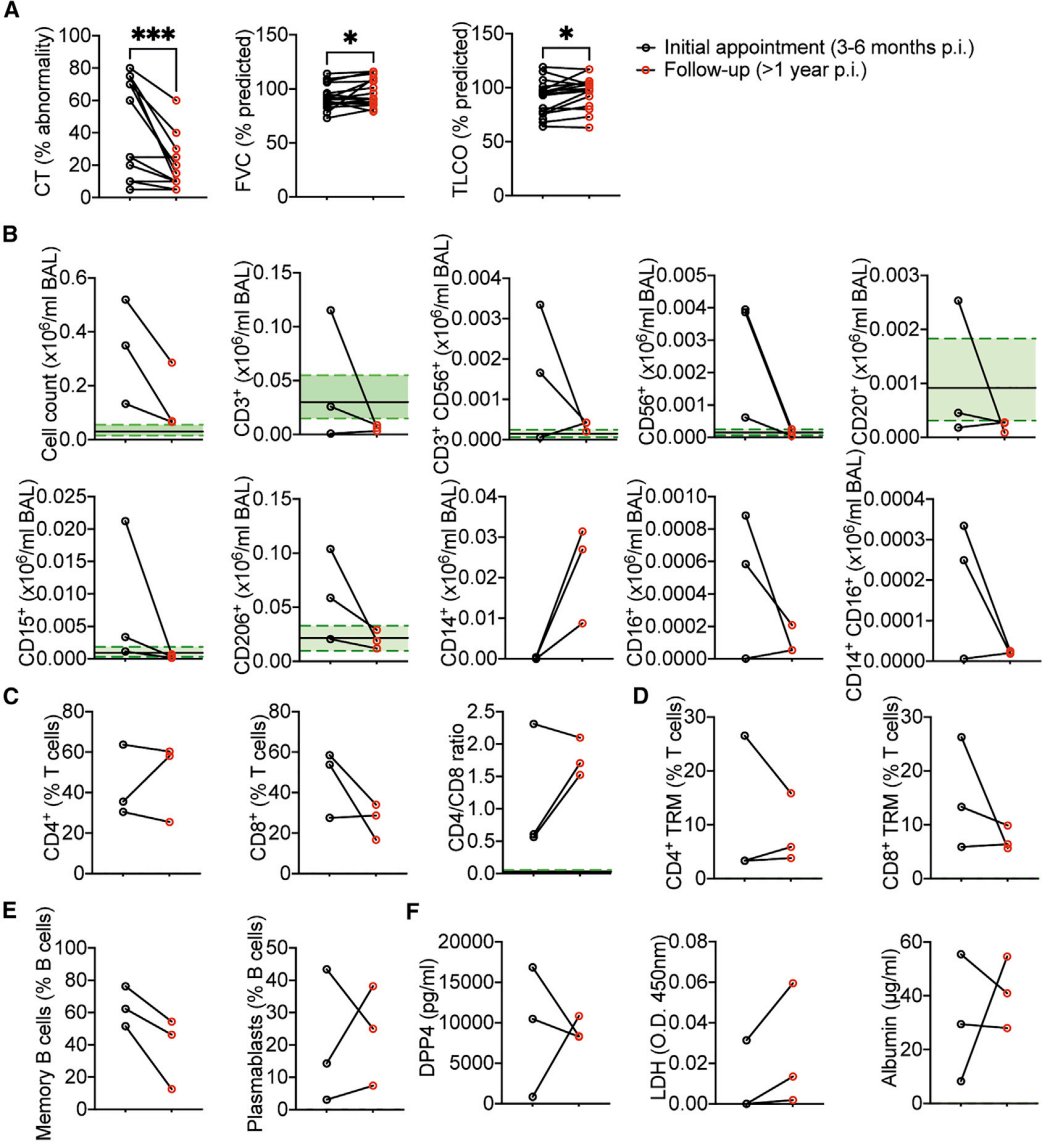
Reduced cellularity is observed in the airways 1 year after initial bronchoscopy post-COVID-19 (A) % lung CT abnormality or predicted FVC (%) or TLCO (%) at first appointment and 1 year follow up (n = 17 pCOVID-19 patients). (B) Total cell counts and cell counts of lymphocyte populations, macrophages, neutrophils, and monocyte subsets in the BAL. (C and D) (C) Proportions of T cell subsets and (D) CD4 and CD8 CD69^+^ CD103^+^ as a proportion of BAL T cells. (E) Proportions of memory (CD27^+^IgD^−^) and plasmablasts (CD27^+^CD38^+^) of CD20^+^ B cells in the BAL. (F) DPP4, LDH, and albumin measurements in BAL. All data depict first bronchoscopy between 3–6 months post discharge and at 1 year post discharge. Each point represents a single patient. (B–E) Represent n = 3 patients. Green shading indicates median ± IQR for proportions of populations and mediator concentration observed in healthy airways. (A) Wilcoxon matched-pair signed rank test. ^∗^p < 0.05, ^∗∗∗^p < 0.001.

A subset of our cohort, who initially became infected with SARS-CoV-2 in Spring 2020, were also clinically assessed at 1 year post discharge. In line with a larger study, which included individuals without clinical indications requiring a bronchoscopy of radiological changes post-COVID-19 (Vijayakumar et al., 2021), substantial reductions in CT abnormality within the lungs were seen at 1 year post discharge, compared with 3–6 months post discharge (Figure 7A). Improvements were also seen in patients’ predicted FVC and TLCO by 1 year post discharge (Figure 7A).

There was, however, some variation in the degree of improvement from post-COVID-19 respiratory disease, and three of the patients examined at 1 year post discharge continued to have substantive lung CT abnormalities, justifying a follow-up bronchoscopy (demographics presented in Table S3). The total number of BAL cells recovered was greatly reduced in all three patients between the initial bronchoscopy and the 1-year follow-up bronchoscopy, and comparable to HC airways (Figure 7B). Similarly, numbers of T, B, NK, and NKT cells along with neutrophils and AMs were reduced to nearly or within the normal range seen in the airways of healthy individuals (Figure 7B). Non-classical and intermediate monocytes were also reduced at 1 year post discharge, but classical monocytes increased (Figure 7B). In the two individuals with elevated lymphocytes, the ratio of CD4 to CD8 T cells increased (Figure 7C). Moreover, the proportion of CD8, but not CD4, T cells trended to decrease, although the proportion of each that were of a Trm or activated (CD69^+^) phenotype remained similar between the two time points (Figure 7D). Memory B cell proportions, but not plasmablasts, also declined between 3 and 6 months and 1 year post discharge (Figure 7E). Fitting with a progressive recovery trajectory, airway DPP4 concentrations declined in the two patients with elevated concentrations at the first bronchoscopy (Figure 7F). However, it is notable that LDH, which was low to non-detectable in all three patients at the first bronchoscopy, showed a trend to increase, while albumin concentrations were unchanged but also within the range of HCs at both time points (Figure 7F).

Collectively, our findings show ongoing changes to the immune and proteomic landscape of the airways. Distinct immune-protein signatures are associated with different pathophysiological changes in the post-COVID-19 lung. However, these changes, and lung pathology, do appear to resolve over the longer (>1 year) term.

## Discussion

Recovery from COVID-19 may be complicated by long-lasting symptoms, including breathlessness. Here, we studied patients previously hospitalized with COVID-19, revealing persistent proteomic and immunological abnormalities in the airways, but not the peripheral blood, many months after acute infection. While there is substantial heterogeneity between patients, we observed upregulation of proteins associated with ongoing cell death, epithelial damage, and tissue repair in post-COVID-19 airways. This correlated with the presence of increased numbers of activated tissue-resident CD8 T cells. Preliminary evidence suggests that this altered airway landscape does improve over the long term, with reductions in airway immune cell numbers 1 year post discharge.

The acute response to SARS-CoV-2 infection is characterized by widespread upregulation of circulating proteins, including IFN pathway proteins, chemokines, cytotoxic proteins, and markers of epithelial damage (Arunachalam et al., 2020; Filbin et al., 2021; Gisby et al., 2021). More severe disease is associated with increased inflammatory proteins (e.g., IL-6, TNF, GM-CSF, IL-1RN, and IL-18) (Arunachalam et al., 2020; Filbin et al., 2021; Thwaites et al., 2021). A similar pattern of upregulated proteins, especially chemokines such as CXCL10, and cytokines such as IL-6, is seen in the airways during acute COVID-19 (Liao et al., 2020; Saris et al., 2021; Szabo et al., 2021). However, 3–6 months after SARS-CoV-2 infection, despite the presence of ongoing respiratory morbidity, the plasma proteins differentially expressed during acute disease appear to have returned to similar concentrations to those seen in HCs. Even data dimension reduction approaches such as WGCNA fail to highlight any significant associations between COVID-19 infection and the plasma proteome months later.

In contrast, the post-COVID-19 airways continue to display an abnormal proteome, with both distinct and shared features to that seen in acute disease. Proteins linked to inflammation feature less prominently than in acute COVID-19, whereas upregulation of proteins involved in epithelial damage and repair (e.g., the EGFR ligand AREG and the epithelial marker KRT19) persist. MMP-3, which regulates the ECM, was also differentially upregulated in the post-COVID-19 airway. MMP3 and AREG are both upregulated after influenza A virus (IAV) infection *in vivo* in mice, and *in vitro* in human fibroblasts and epithelial cells (Boyd et al., 2020), and both are linked to epithelial repair and fibrosis in the lungs (Morimoto et al., 2018; Yamashita et al., 2011).

Elevated LDH and albumin in the airways provides further evidence of ongoing cell death and damage to respiratory barrier integrity post-COVID-19. This observation is reinforced by the upregulation of a module of correlated proteins in the post-COVID-19 BAL, whose individual members reflect epithelial damage (EPCAM, KRT19), cell death (CASP3), and epithelial repair (TGFA) but also suggest a connection between these processes and immune cell recruitment and survival (CXCL9, CXCL10, CXCL11, IL-7). Increased cell death within the airways correlates with the frequency of T cells, primarily CD8 Trm cells, and with heightened respiratory dysfunction. In mouse models of severe acute respiratory virus infection, CD8 T cells are known to act as a double-edged sword. Although the cytotoxic molecules and cytokines they release are essential for clearing the virus, they can also cause tissue damage and immunopathology (reviewed in Duan and Thomas [2016] and Schmidt and Varga [2018]). While pre-existing virus-specific CD8 Trm cells in the airways are thought to be protective against a re-encounter with the same virus (Jozwik et al., 2015; Wu et al., 2014), little is known about their role in long-term respiratory virus-related pathology, especially in humans. This is primarily due to the lack of relevant samples collected during the recovery period. Our post-COVID-19 data support the concept that sustained activation of CD8 Trm cells in the airways long after recovery from acute disease contributes to the ongoing damage to the respiratory epithelium, resulting in airway disease.

The mechanism underlying increased Trm cells in the airways is unclear, although several studies have reported virus-specific CD8 T cells in lung tissue up to a year post-infection (Cheon et al., 2021; Grau-Expósito et al., 2021; Poon et al., 2021). While virus-specific CD4 and CD8 T cells rapidly expand and form Trm cells following SARS-CoV-2 infection (Szabo et al., 2021), these cells rapidly contract after the resolution of acute disease, with CD8 Trm cells declining more rapidly than CD4 Trm cells (Slütter et al., 2017). However, the lungs of mice which previously experienced IAV infection maintain CD8 Trm cells more robustly compared with uninfected lungs, showing that severe infection promotes a pro-Trm niche (Slütter et al., 2017). This fits with our observation that CD8 Trm cell numbers vary dependent on the proteins and extent of damage in the airways, and change longitudinally in the same individuals, while CD4 Trm cells remain relatively static. A number of factors may contribute to the heterogeneity of the CD8 Trm niche in the post-COVID-19 airway. First, while all our post-COVID-19 samples were taken from patients who tested negative for SARS-CoV-2 by qPCR immediately prior to bronchoscopy, persistent antigens have been observed months after other respiratory infections such as IAV (Kim et al., 2010), and SARS-CoV-2 antigen depots could drive ongoing cytotoxic activity and maintenance of CD8 Trm cells. Second, the persistence of lung-resident Trm cells is reliant on the availability of local T cell survival signals such as IL-7 (Szabo et al., 2019) and the CXCR3 ligands (Slütter et al., 2013). Indeed, IL-7 and the CXCR3 ligands are part of the protein network that is maintained in the post-COVID-19 airway. Lastly, there is some evidence for indicating the development of auto-immunity in some patients recently recovered from COVID-19 (Lucas et al., 2020; Wang et al., 2020). It is likely that these different mechanisms collectively act to shape CD8 Trm cell responses and other immune cells in the post-COVID-19 airway, and the scale and duration of ongoing epithelial damage and respiratory dysfunction observed.

B cell frequencies were more elevated in individuals with more widespread lung abnormalities and reduced gas exchange. During acute infection or after vaccination, B cells are critical in the generation of protective virus-specific antibody. Virus-specific B cells can be detected in the lungs up to 6 months post SARS-CoV-2 infection (Poon et al., 2021) but represent a minority of the B cells present in the human lung. Increased frequencies of airway and lung B cells, similar to those seen in the post-COVID-19 airway, are commonly seen in a range of respiratory diseases including COPD and interstitial lung diseases (ILDs) (Desai et al., 2018; Polverino et al., 2016). B cell frequencies do not correlate with virus-specific antibodies, which are more tightly linked to the T cell responses, suggesting a common antigen specific driver that B cells are not dependent on. Precisely how B cells contribute to ongoing respiratory pathology post-COVID-19 is unclear; they can produce both pro-inflammatory and regulatory factors, and disruption of regulatory B cell function has been shown to be associated with fibrotic lung disease (Asai et al., 2019). B cells can also promote tissue repair by inducing activation and migration of fibroblasts (Ali et al., 2021). Thus, in the post-COVID-19 airway, B cells may be directly promoting aberrant tissue repair.

Functional impairment of monocytes and DCs in the peripheral blood of acutely infected patients (Arunachalam et al., 2020; Laing et al., 2020; Mann et al., 2020), as well as hyperactivation of airway monocyte populations, are features of acute severe COVID-19 (Liao et al., 2020; Szabo et al., 2021). In our post-COVID-19 patients, peripheral blood monocytes had normalized and did not correlate with markers of pulmonary dysfunction, but BAL intermediate monocytes were increased in patients with greater CT abnormalities. In humans, following inflammatory insults, monocytes are recruited to the airways to differentiate into new AMs (Byrne et al., 2020). Severe viral infection can cause rapid depletion of the AM pool (Pribul et al., 2008), and different subsets of monocytes contribute differentially to the replenishment of lung macrophages (Evren et al., 2021). Monocyte to macrophage transition is also more pronounced in chronic lung disease, with the newly generated monocyte-derived macrophages acting in a pro-fibrotic fashion (Misharin et al., 2017). Increases in intermediate monocytes may therefore be indicative of heightened monocyte differentiation into AMs, the numbers of which are increased in the post-COVID-19 airway compared with HCs. Amplification of this process may then contribute to ongoing repair within the lungs.

The progressive resolution of radiological abnormalities in the majority of post-COVID-19 patients has been described (Han et al., 2021), and in our study even the three patients with persistent respiratory abnormalities showed improved CT and reduced airway immune cell infiltration. This fits with the hypothesis that SARS-CoV-2 infection can result in organizing pneumonia, with subsequent changes reflecting ongoing epithelial damage and healing parenchyma rather than established fibrosis (Kory and Kanne, 2020). Moreover, the involvement of the immune response in different aspects of ongoing respiratory disease post-COVID-19 suggests that this recovery could be accelerated using immunomodulatory treatments.

### Limitations of the study

Our post-COVID-19 data are generated on patients undergoing clinically indicated bronchoscopy because of persistent respiratory abnormalities. Whether our findings extend to individuals with no radiological abnormalities or respiratory symptoms post-COVID-19 remains unknown. This selection bias also affects longitudinal sampling greater than 12 months post-COVID-19, since the majority of patients initially sampled between 3 and 6 months post-COVID-19 had shown sufficient improvement in respiratory pathology such that a follow-up bronchoscopy was not indicated.

Although we did not detect a plasma proteomic signature post-COVID-19, our limited sample size is likely not powered to detect small differences in circulating proteins between post-COVID-19 patients and HCs. Examination of p values distribution suggests that differences may exist but will require much larger studies to reveal them. Regardless, the absence of any correlation between the differentially expressed proteins in the airways and their corresponding changes in the plasma points to the limited utility of peripheral blood as an indicator of the pathological lung processes. A limitation of the Olink platform used is that the proteins measured were highly enriched for those involved in immuno-inflammatory processes, and thus we did not have an unbiased assessment of the entire proteome.

Finally, as with most studies, we were limited to sampling the airways post-infection and did not have paired pre-infection samples for intra-individual comparisons. It is possible, therefore, that some differences observed between HCs and post-COVID-19 patients could reflect a pre-infection phenotype. Indeed, one of the most differentially expressed proteins in the airways, DPP4, is the binding receptor for another coronavirus MERS (Raj et al., 2013) and is capable of mediating some SARS-CoV-2 binding (Li et al., 2020). Thus, it is conceivable that pre-existing upregulation of DPP4 increased susceptibility to post-COVID-19 syndrome via increased viral entry (i.e., reverse causation), rather than DPP4 upregulation occurring in response to COVID-19. However, the longitudinal reduction of DPP4, alongside reduced CT abnormalities and increased pulmonary function, argues against this hypothesis. More generally, the majority of proteins and markers upregulated are associated with ongoing lung pathology in other contexts (e.g., LDH) and are absent or only present at very low concentrations in healthy airways, suggesting that their upregulation is more likely to be a consequence of COVID-19 than a pre-disposing risk factor.

## STAR★Methods

### Key resources table

**Table:** 

REAGENT or RESOURCE	SOURCE	IDENTIFIER
**Antibodies**		

Anti-Human CD69, BUV395	BD Biosciences	Cat#564364; RRID: AB_2738770
Anti-human CD8, BUV496	BD Biosciences	Cat#612942; RRID: AB_2870223
Anti-Human CD45RA, BUV563	BD Biosciences	Cat#612927; RRID: AB_2870212
Anti-Human CD11c, BUV661	BD Biosciences	Cat#612968; RRID: AB_2870241
Anti-Human CD56, BUV737	BD Biosciences	Cat#612767; RRID: AB_2860005
Anti-Human CD3, BUV805	BD Biosciences	Cat#612896; RRID: AB_2870184
Anti-Human IgD, BV421	Biolegend	Cat#348226; RRID: AB_2561619
Anti-Human CD16, SuperBright436	ThermoFisher	Cat#62-0166-42; RRID: AB_2716985
Anti-Human CD25, eFluor450	ThermoFisher	Cat#48-0257-42; RRID : AB_2574011
Anti-Human CD20, BV480	BD Biosciences	Cat#566132; RRID: AB_2739531
Anti-Human CD127, BV510	Biolegend	Cat#351332; RRID: AB_2562304
Anti-Human HLA-DR, BV570	Biolegend	Cat#307638; RRID: AB_2650882
Anti-Human CD28, BV605	Biolegend	Cat#302968; RRID: AB_2800755
Anti-Human CD38, BV650	Biolegend	Cat#356620; RRID: AB_2566233
Anti-Human CD15, BV711	Biolgend	Cat#323050; RRID: AB_2750192
Anti-Human CD279, BV750	Biolegend	Cat#329966; RRID: AB_2810505
Anti-Human CD206, BV785	Biolegend	Cat#321142; RRID: AB_2734302
Anti-Human CD45, QDOT800	ThermoFisher	Cat#Q10156; RRID: AB_1500477
Anti-Human CXCR5, BB515	BD Biosciences	Cat#564624; RRID: AB_2738871
Anti-Human CD169, AF488	R&D systems	Cat#FAB5197G; RRID: AB_2905550
Anti-Human CD4, Spark Blue 550	Biolegend	Cat#344656; RRID: AB_2819979
Anti-Human CD161, PerCP	Biolegend	Cat#339933;RRID: AB_2563998
Anti-Human CD27, BB700	BD Biosciences	Cat#566449; RRID: AB_2739731
Anti-Human Siglec8, PerCP Cy5.5	Biolegend	Cat#347108; RRID: AB_2629716
Anti-Human CD86, PerCP eFLuor710	ThermoFisher	Cat#46-0869-42; RRID: AB_10596362
Anti-Human CD141, PE	Biolegend	Cat#344104; RRID: AB_2255842
Anti-Human TCRg/d, PEdz594	Biolegend	Cat#331226; RRID: AB_2565534
Anti-Human TCRa/b PE Cy5	Biolegend	Cat#306710; RRID: AB_314648
Anti-Human CD11b, PE Cy7	Biolegend	Cat#301322; RRID: AB_830644
Anti-Human CD123, APC	Biolegend	Cat#306012; RRID: AB_439779
Anti-Human CRTH2, AF647	Biolegend	Cat#350104; RRID: AB_10642025
Anti-Human CD14, Spark NIR	Biolegend	Cat#367150; RRID: AB_2820023
Anti-Human CD1c, APC R700	BD Biosciences	Cat#566614; RRID: AB_2869794
Anti-Human CD103, APC Cy7	Biolegend	Cat#350228; RRID: AB_2734362
Anti-Human CD45, PerCP Cy5.5	ThermoFisher	Cat#45-0459-42; RRID: AB_10717530
Anti-Human Siglec8, AF488	R&D systems	Cat#FAB7975G; RRID: AB_2905535
Anti-Human CD19, BV421	Biolegend	Cat#302234; RRID: AB_11142678
Anti-Human CD4, BV510	Biolegend	Cat#317444; RRID: AB_2561866
Anti-Human CD117, BV605	Biolegend	Cat#313218; RRID: AB_2562025
Anti-Human CD14, BV711	Biolegend	Cat#301838; RRID: AB_2562909
Anti-Human CD16, BV785	Biolegend	Cat#302046; RRID: AB_2563803
Anti-Human CD177, FITC	Biolegend	Cat#315804; RRID: AB_2072603
Anti-Human Siglec8, PE	R&D systems	Cat#FAB7975P; RRID: AB_2905537
Anti-Human CD56, PEdz594	Biolegend	Cat#318348; RRID: AB_2563564
Anti-Human CD3, PE-Cy7	Biolegend	Cat#300420; RRID: AB_439781
Anti-Human CD206, APC	Biolegend	Cat#321110; RRID: AB_571885
Anti-Human FcE, AF700	Biolegend	Cat#334630; RRID: AB_2571902

**Chemicals, peptides, and recombinant proteins**		

LIVE/DEAD Fixable NIR Cell Stain	ThermoFisher	Cat#L34976
LIVE/DEAD Fixable Blue? Cell Stain	ThermoFisher	Cat#L34961
TruStain FcX	Biolegend	Cat#422302
RPMI 1640	Gibco	Cat#21875091

**Critical commercial assays**		

Target 96 Cardiometabolic Assay	Olink	Cat#91802
Target 96 CVD II Assay	Olink	Cat#91202
Target 96 CVD III Assay	Olink	Cat#91203
Target 96 Immune Response Assay	Olink	Cat#91701
Target 96 Inflammation Assay	Olink	Cat#91301
DPP4 ELISA	R&D Systems	Cat#DY1180
Albumin ELISA	Bethyl Laboratories	Cat#E80-129
LDH Assay	Sigma Aldrich	Cat#TOX7
LEGENDplex Human Proinflam. Chemokine Panel 1	Biolegend	Cat#740984

**Deposited data**		

Proteomic data and associated clinical and demographic information	Dryad	https://doi.org/10.5061/dryad.2ngf1vhq3
R code used in analysis of proteomic data	Github/Zenodo	https://doi.org/10.5281/zenodo.5844957

**Software and algorithms**		

Flowjo version 10.7 software	Treestar	https://www.flowjo.com
STRING protein module visualisation	String	https://www.string-db.org
RStudio version 1.2.1335	RStudio	https://www.rstudio.com
R version 3.5	R Foundation for Statistical Computing	https://www.R-project.org

### Resource availability

#### Lead contact

Further information and requests for resources and reagents should be directed to and will be fulfilled by lead author James A. Harker (j.harker@imperial.ac.uk).

#### Materials availability

This study did not generate new unique reagents.

### Experimental model and subject details

#### Human samples

Post-COVID19 bronchoalveolar lavage fluid (BAL) was obtained from patients recruited to the PHENOTYPE study (NCT 04459351), an observational, longitudinal study recruiting patients at Chelsea and Westminster Hospital, London. 38 samples were collected from patients requiring sampling for clinical purposes. Ethical approval for the study was given by Yorkshire & The Humber - Sheffield Research Ethics Committee (IRAS 284497).

Patients who met the inclusion and exclusion criteria were recruited to the PHENOTYPE study (demographics in Table S1):

Inclusion criteria for the study were:

• Aged 18 years or older
• Previous confirmed COVID-19 infection (positive PCR or antibody)
• Attending a respiratory follow-up outpatient appointment for follow-up of persistent respiratory symptoms following visit post hospital attendance with COVID-19. infection or referred by the community for covid-related symptoms.

Patients were seen at approximately 4-6 weeks (Visit 1) and 3 months (Visit 2) following discharge from hospital or referral (if referred from the community). Patients underwent clinical assessment at both visits, including collection of demographic data, clinical history and clinical examination and assessment of vital parameters (heart rate, peripheral oxygen saturations, blood pressure reading and temperature). They also underwent clinical blood tests (including full blood count, renal function, liver function, C-reactive protein (CRP), ferritin, fibrinogen, D-dimer and pro-calcitonin). Patients had a computed tomography (CT) scan of the lungs approximately 3 months post discharge from hospital. In patients with abnormal CT findings, or persistent respiratory symptoms, a bronchoscopy and lavage was performed as part of clinical work-up. Bronchoscopy was performed under conscious or deep sedation. 150 ml of normal saline were instilled into the most affected segment (as determined by CT imaging), in 50 ml aliquots. 10 ml of fluid return was used for the scientific analysis described in this paper. Patients underwent formal lung function tests (including spirometry, lung volumes and gas transfer) near the time of the bronchoscopy (usually during the days immediately preceding the procedure). Lung function testing was performed in accordance with the American Thoracic Society and European Respiratory Society guidelines (2019). Further follow-up was determined on the basis of clinical need, with a maximum follow up period of up to 2 years post hospital discharge or referral.

Control, uninfected bronchoalveolar lavage was obtained from healthy donors (collected between April 2016 and December 2019). Ethical approval for the study was granted by the Research Ethics Committee (15/SC/0101) and all patients provided informed written consent as described previously (Allden et al., 2019; Byrne et al., 2020; Invernizzi et al., 2021). Briefly, 240 ml aliquots of warmed sterile saline were instilled in the right middle lung and aspirated by syringe. Lavage aliquots were pooled for each subject. All subjects provided written, informed consent to participate in the study. Healthy volunteers had no self-reported history of lung disease, an absence of infection within the last 6 months and normal spirometry.

### Method details

#### Scoring of Computed Tomography scans

All CT scans were reviewed by two Thoracic Radiologists (AD and SRD), who have over 20 years’ experience, and were blinded to the clinical data. CT scans were scored by consensus and the overall extent of opacified lung quantified to the nearest 5%.

#### Processing of airway bronchoalveolar lavage samples

BAL samples were processed and stained on the day of sample collection. BAL was strained through a 70μm filter and subsequently centrifuged (1800 rpm, 2 min, 4**°**C). Supernatant was snap-frozen and stored at -80**°**C. Pellets were incubated in red blood cell lysis buffer (155mM NH_4_Cl, 10mM KHCO_3_, 0.1mM ethylenediaminetetraacetic acid, pH 7.4) for 10 minutes before washing and resuspension in complete media (RPMI 1640 with 10% fetal calf serum, 2mM L-glutamine, 100U/ml penicillin-streptomycin).

#### Processing of blood samples

Peripheral blood was collected in EDTA coated vacutainers on the same day as bronchoscopy. 1ml blood was centrifuged at 100g for 10 minutes (4**°**C), followed by centrifugation at 20,000g for 20 minutes (4**°**C) to separate plasma, which was subsequently stored at -80**°**C. 2ml blood from post-COVID-19 patients was incubated with red blood cell lysis buffer (155mM NH_4_Cl, 10mM KHCO_3_, 0.1mM ethylenediaminetetraacetic acid, pH 7.4) for 10 minutes before washing and resuspension in complete media (RPMI 1640 with 10% fetal calf serum, 2mM L-glutamine, 100U/ml penicillin-streptomycin). 2.5 ml blood from healthy controls was used to isolate peripheral blood mononuclear cells (PBMC) by Percoll density centrifugation, as per manufacturer’s instructions.

#### Flow cytometry staining

For traditional flow cytometry, 2 - 5 x10^5^ cells were plated, while for high parameter analysis using the Cytek Aurora 1 x 10^6^ cells from each site were used. Cells were washed with PBS and incubated with either near-infrared (traditional flow cytometry) or blue (Cytek Aurora) fixable live/dead stain (Life Technologies), as per the manufacturer’s instructions. Before incubation with human fc block (BD Pharmingen) cells were washed with FACS buffer (1% FCS, 2.5% HEPES, 1mM EDTA) and surface staining was performed at 4**°**C for 30 minutes using antibody panels as described in the key resources table. Surface staining was followed by washing with FACS buffer and fixation with 1% paraformaldehyde for 10 minutes. Labelled cells were acquired on a 4-laser BD Fortessa (traditional flow cytometry; BD Bioscience) or 5-laser Cytek Aurora flow cytometer (Cytek Bio).

#### Flow cytometry analysis

Conventional flow cytometry data was analysed using FlowJo v 10.6 (Tree Star). Data was pre-gated to exclude doublets and dead cells. In BAL samples CD45^+^ cells were selected, and immune cell populations were identified using the gating strategy shown in Figure S1A. Percentages of the CD45^+^ gate were calculated. In blood samples, leukocytes were selected based on FSC and SSC and immune cell populations were identified using the gating strategy shown in Figure S1A. Percentages of total leukocytes were calculated. High-parameter spectral deconvolution flow cytometry data from the Cytek Aurora was analysed using Cytobank (Beckman). tSNE analysis was performed on 300,000 events from 11 files. Iteration number was set to 1500 with a perplexity of 30 and theta of 0.5. FlowSOM analysis was performed subsequently using hierarchical consensus clustering with 12 metaclusters, 100 clusters and 10 iterations. Manual gating of high parameter cytometry data was carried out as shown in Figure S6A. Heatmaps were generated from median fluorescence values in Prism 9.0 (GraphPad).

#### Olink proximity extension proteomic assay

Plasma and BAL proteomic measurement was performed using the Olink proximity extension immunoassay platform. Five 92-protein multiplex Olink panels were used (‘Inflammation’, ‘Immune Response’, ‘Cardiometabolic’, Cardiovascular 2’, ‘Cardiovascular 3’), providing measurements of 460 protein targets per sample. Cryopreserved BAL and plasma samples were thawed on ice and mixed well by pipetting before plating 88 samples per plate ensuring case/control balance and random well ordering to prevent confounding of technical and biological effects. For BAL samples, a pilot study was performed using three control samples and three post-COVID19 samples (severe group) to determine optimal dilution parameters. Ultimately BAL was used neat. Since a small number of proteins were assayed on more than one panel, we measured a total of 435 proteins. We removed duplicate assays at random prior to subsequent analyses.

#### CXCR3 chemokine composite score

To create a composite score that reflected the CXCR3 chemokines (CXCL9, CXCL10 and CXCL11), we used the following approach. For each sample, protein concentration for CXCL9, -10 and -11, were normalized to the median concentration in healthy controls (to avoid unduly weighting the score towards chemokines with higher NPX values). For each sample, the mean of the normalized values for the 3 proteins was then calculated to provide a summary metric for CXCR3 chemokines.

#### Epithelial damage marker analysis in BAL

DPP4 (R&D systems, DY1180) and albumin (Bethyl Laboratories, E80-129) concentrations in the BAL were quantified by ELISA according to manufacturer’s instructions. LDH concentrations were quantified using an *in vitro* toxicology assay (Sigma, TOX7). Briefly, 25μl of BAL sample were incubated with 50μl of LDH assay reaction mixture. After 30 minutes, the reaction was stopped with 7.5μl 1N HCL and absorbance was measured at 490nm with background correction at 690nm. All absorbances were measured using a SpectraMax i3x (Molecular Devices).

#### Total antibody measurement in BAL

Total antibody concentrations were measured in BAL by ELISA according to manufacturer’s instructions (ThermoFisher Scientific, 88-50550-88, 88-50600-88). Briefly, plates were coated overnight with anti-IgG or –IgA capture antibody. BAL samples were added to plate at a dilution of 1:500 for IgG and 1:100 for IgA and incubated for 2 hours at room temperature. Plates were next incubated with detection antibody for 1 hour at room temperature and developed with TMB substrate. Absorbances at 450nm were measured using a SpectraMax 3i plate reader (Molecular Devices, USA)

#### SARS-CoV-2 RBD-specific antibody measurement

ELISAs against RBD-specific IgG and IgA were developed in-house using recombinant SARS-CoV-2 spike RBD protein (Sino Biologicals Inc., 40592-VNAH). Plates were coated overnight with 1μg/ml of protein and BAL and plasma samples were serially diluted from neat and 1:20, respectively, and incubated at room temperature for 2 hours. Pooled plasma samples from positive controls were added to each plate to allow for normalisation. Plates were incubated with goat anti-human IgG/IgA-HRP (Southern Biotech, 2040-05/2050-05) for 1 hour at room temperature. Plates were developed with TMB substrate (Neogen, 308177) and reactions stopped with 0.18M sulfuric acid before measurement of absorbance at 450nm using a SpectraMax 3i plate reader (Molecular Devices, USA).

#### Pro-inflammatory chemokine analysis in BAL

13 pro-inflammatory chemokines were measured in BAL using a LEGENDplex bead-based assay according to manufacturer’s instructions (Biolegend, 740984). Briefly, 25μl of neat BAL sample was added to 25μl assay buffer. Beads were added to each well and incubated on a shaker at 800rpm for 2 hours at room temperature. Plates were centrifuged and washed before addition of detection antibody. Plates were incubated with detection antibody for 30 minutes on a shaker at 800rpm. Plates were washed and samples were acquired using a BD Fortessa flow cytometer (BD Biosciences, USA). Data were analysed using the LEGENDplex data analysis software (Biolegend).

### Quantification and statistical analysis

#### Olink proximity extension proteomic analyses

Proteomic data was normalized using standard Olink workflows to produce relative protein abundance on a log_2_ scale (‘NPX’). BAL and plasma proteomic data were normalized separately. Quality assessment was performed by (1) examination of Olink internal controls and (2) inspection of boxplots, relative log expression plots, and PCA.

PCA was performed using singular value decomposition. Following these steps, 2 clear outlying samples were removed from the BAL dataset. To identify proteins that were differentially abundant between case and controls, for each protein we performed linear regression (lm function in R) with case/control status as the independent variable and protein concentration (NPX/ml) as the dependent variable. P-values were adjusted for multiple testing using the Benjamini-Hochberg procedure (p.adjust function in R). A 5% false discovery rate was used to define statistical significance. We used the WGCNA R package (Langfelder and Horvath, 2008; Zhang and Horvath, 2005) to create a weighted protein correlation network. Prior to WGCNA analysis, protein data were scaled and centred, and missing data were imputed using the R caret package. We used the WCGNA adjacency function to produce a weighed network adjacency matrix, using parameters “type=signed” and “power=13”. This soft-thresholding power was selected as the lowest power to achieve approximate scale-free topology. We next defined a topological overlap matrix of dissimilarity using the TOMdist function. Clusters (‘modules’) of interconnected proteins were identified using hierarchical clustering and the cutreeDynamic function with parameters: method=“hybrid”, deepSplit=2, minClusterSize=15. We then tested association of these modules with case/control status. Multiple testing correction was performed to account for the number of modules. We report both Benjamini-Hochberg and Bonferroni adjusted p-values to provide two levels of stringency. To assess the distribution of p-values from the differential protein abundance analyses, we plotted histograms and constructed QQ plots. QQ plots were made by comparing the expected distribution of -log_10_ P values under the null hypothesis of no proteomic differences between post-COVID19 patients and controls to the observed p-values for the 435 proteins. We performed pathway enrichment analysis for the 435 proteins measured. This was performed using terms from KEGG database (Table S2B) and the Reactome database (Table S2C). Protein modules were visualised using STRING (https://string-db.org/), with known or suspected interconnections between module members displayed as edges in a network diagram. An edge represents a protein-to-protein relationship defined as shared contributions to a particular function, and not necessarily implying physical binding. In Figure 3C, edge colour indicates the type of evidence for the relationship: turquoise represents known interactions from curated databases; magenta represents experimentally determined interactions; green represents predicted Interactions from gene neighbourhood analysis; red represent predicted interactions from gene fusions, blue represent predicted Interactions from gene co-occurrence; light green represents interaction from text-mining; black represents interaction from co-expression data, and violet represents information from protein homology.

#### Quantification and statistical analysis for flow cytometry and univariate assays

Differences in means between two sample groups were compared using two-tailed Mann-Whitney U tests. Multiple group comparisons were done using Kruskal Wallis ANOVA followed by Dunn’s post-test. Spearman-Rank correlations immune cell versus clinical and biomarker traits. Analysis was performed in GraphPad Prism. For all figures, ^∗^ denotes p value < 0.05, ^∗∗^ denotes p value < 0.01 and ^∗∗∗^ denotes p value < 0.001. Where multiple tests were carried out significance was assessed by carrying out a Benjamini-Hochberg set to 5% FDR.

#### Summary diagrams

The summary schematic and graphical abstract were designed using BioRender.

## Data Availability

Proteomic data has been deposited in the Dryad repository and are publicly available as of the date of publication. Accession numbers are listed in the key resources table. All original code has been deposited at Zenodo and is publicly available as of the date of publication. Additional Supplemental Items (Table S2) are available from Mendeley Data: https://doi.org/10.17632/th35tt4zwm.1. All DOIs are listed in the key resources table.

## Supplemental information

Document S1. Figures S1–S7 and Tables S1 and S3

Table S2. Post-COVID-19 proteomics analysis, related to Figure 3 A spreadsheet (tables A–L) detail statistical analysis of post-COVID-19 BAL and plasma proteomes. Doi:10.17632/th35tt4zwm.1.

Document S2. Article plus supplemental information
