# Supplementary material for: Immuno-proteomic profiling reveals aberrant immune cell regulation in the airways of individuals with ongoing post-COVID-19 respiratory disease
Source: Immunity. 2022 Mar 8;55(3):542–556.e5. doi: 10.1016/j.immuni.2022.01.017 (PMC8789571; doi:10.1016/j.immuni.2022.01.017)
Supplement: Document S1. Figures S1–S7 and Tables S1 and S3 [file mmc1.pdf]

Immunity, Volume *55*

Supplemental information

Immuno-proteomic profiling reveals aberrant immune cell regulation in the airways of individuals with ongoing post-COVID-19 respiratory disease

Bavithra Vijayakumar, Karim Boustani, Patricia P. Ogger, Artemis Papadaki, James Tonkin, Christopher M. Orton, Poonam Ghai, Kornelija Suveizdyte, Richard J. Hewitt, Sujal R. Desai, Anand Devaraj, Robert J. Snelgrove, Philip L. Molyneaux, Justin L. Garner, James E. Peters, Pallav L. Shah, Clare M. Lloyd, and James A. Harker

Table S1: Demographics of the post-COVID19 and healthy control cohort. Related to Figure 1. Abbreviations: Gender, M (male), BAME (black, Asian and minority ethnic), COPD (chronic obstructive pulmonary disease), CPAP (continuous positive airway pressure), NIV (Non-invasive ventilation), IMV (invasive mechanical ventilation), CT (computed tomography), FEV1 (forced expiratory volume in 1 second), FVC (forced vital capacity), TLC (total lung capacity), TLCO (transfer factor of the lung for carbon monoxide), KCO (carbon monoxide transfer coefficient. Data presented as n (%n), median (IQR)

	Post-COVID19 (n = 38)	Healthy controls (n = 29)
Age (years)	56.5 (51.3-66.0)	49.0 (39.5 - 54.5)
Gender, M (%n)	30 (78.9)	17 (58.6)
BAME (%n)	22 (57.8)	N/A
Comorbidities (%n)		
Asthma	5 (13.2)	N/A
COPD	1 (2.63)	N/A
Previous pneumothorax	1 (2.63)	N/A
Hypertension	9 (23.7)	N/A
Ischaemic heart disease	3 (7.89)	N/A
Type 2 diabetes mellitus	7 (18.4)	N/A
Smoking history		
Current smoker	1 (2.63)	2 (6.8)
Ex-smoker (>=10 pack year)	5 (13.2)	4 (13.7)
Non-smoker or ex-smoker <10 pack year)	32 (84.2)	17 (58.6)
Severity of acute illness		
Hospitalized (n/%)	36 (94.7)	N/A
- Oxygen therapy only (moderate)	15 (39.5)	N/A
- CPAP/ NIV (severe)	11 (28.9)	N/A
- IMV (very severe)	10 (26.3)	N/A
Not hospitalized (moderate)	2 (5.26)	N/A
Length of hospital stay (days) [n=35]	10.0 (5.0-27.0)	N/A
Admission bloods [n=33]		
White cell count (x10^9/L) [n=33]	6.80 (5.70-8.85)	N/A
Haemaglobin (g/L) [n=33]	143 (132-154)	N/A
Platelet (x10^9/L) [n=33]	219 (195 – 271)	N/A
Neutrophil (x10^9/L) [n=33]	5.90 (3.75-7.30)	N/A
Lymphocyte (x10^9/L [n=33]	1.0 (0.70 – 1.45)	N/A
D dimer (microgram/L) [n=25]	965 (800-1622)	N/A
Fibrinogen (g/L) [n=31]	7.23 (6.21-8.09)	N/A
Ferritin (micrograms/L) [n=26]	762 (606-2329)	N/A
Sodium (mmol/L) [n=33]	134 (133-137)	N/A
Potassium (mmol/L) (n=31)	4.30 (4.1– 4.70)	N/A
Urea (mmol/L) [n=33]	5.30 (3.40-7.10)	N/A
Creatinine (micromol/L) [n=33]	86.0 (75.0-103)	N/A
Egfr (ml/min/1.73m2) [n=33]	79.0 (58.0-90.0)	N/A
Albumin (g/dL) [n=33]	33.0 (30.5-34.0)	N/A
C-reactive protein (mg/L) [n=33]	124 (84.1 – 223)	N/A
CT imaging (n=38)		
Days from admission to follow up CT [n=35]	117 (93.0-141)	N/A
Days from discharge to follow up CT [n=36]	97 (84.3-127)	N/A
Abnormal CT		N/A
Overall CT abnormality		N/A
Ground glass abnormality		N/A
Reticulation		N/A
Consolidation		N/A
Bands		N/A
Bronchoalveolar lavage fluid sampling (n=38)		
Days between admission and sampling [n=35]	153 (107-192)	N/A
Days between discharge and sampling [n=36]	135 (98.5-176)	N/A
Lung function tests [n=36]		
Days between admission and lung function tests [n=33]	147 (106-185)	N/A
Days between discharge and lung function tests [n=34]	118 (92.0-171)	N/A
FEV1 (L)	2.97 (2.36-3.60)	3.26 (2.8 - 3.6)
% predicted FEV1	95.5 (86.5-103)	
FVC (L)	3.73 (2.83 – 4.16)	3.96 (3.5 – 4.9)
% predicted FVC	91.5 (83.5 – 96.8)	
TLC (L)	5.73 (5.09 – 6.33)	N/A
% predicted TLC	89.0 (83.3 – 99.8)	N/A
TLCO ml/min/mmHg	6.39 (5.32 – 8.57)	N/A
% predicted TLCO	78.5 (67.3 – 96.8)	N/A
KCO ml/min/mmHg	1.37 (1.20-1.61)	N/A
% predicted KCO	99.0 (88.3-115)	N/A

Table S3. Demographics, blood tests, CT abnormalities, BALF findings and lung function tests at initial and follow bronchoscopy. Related to Figure 7.

Abbreviations: Gender, M (male), BAME (black, Asian and minority ethnic), COPD (chronic obstructive pulmonary disease), CPAP (continuous positive airway pressure), NIV (Non-invasive ventilation), IMV (invasive mechanical ventilation), CT (computed tomography), FEV1 (forced expiratory volume in 1 second), FVC (forced vital capacity), TLC (total lung capacity), TLCO (transfer factor of the lung for carbon monoxide), KCO (carbon monoxide transfer coefficient

Data presented as n (%n), median (IQR)

	pCOVID (n = 3)
Age (years)	62 (50-71)
Gender, M (%n)	3 (100)
BAME (%n)	2 (66.7)
Comorbidities (%n)	
Asthma	0 (0)
COPD	0 (0)
Previous pneumothorax	0 (0)
Hypertension	3 (0)
Ischaemic heart disease	0 (0)
Type 2 diabetes mellitus	0 (0)
Smoking history	
Current smoker	0 (0)
Ex-smoker (>=10 pack year)	1 (33.3)
Non-smoker or ex-smoker <10 pack year)	2 (66.7)
Severity of acute illness	
Hospitalized (n/%)	3 (100)
- Oxygen therapy only (moderate)	0 (0)
- CPAP/ NIV (severe)	0 (0)
- IMV (very severe)	3 (100)
Not hospitalized (moderate)	0 (0)
Length of hospital stay (days) [n=35]	18 (11-28)
Admission bloods [n=3]	
White cell count ($x10^9$/L)	9.3 (7.4 – 10.3)
Haemaglobin (g/L)	140 (130 – 151)
Platelet ($x10^9$/L)	243 (219 – 524)
Neutrophil ($x10^9$/L)	7.7 (6.1 – 9.1)
Lymphocyte ($x10^9$/L	0.9 (0.7 – 0.9)
D dimer (microgram/L)	1920 (965 – 2874)
Fibrinogen (g/L)	7.5 (7.2 – 8.6)
Ferritin (micrograms/L)	745 (738 – 6032)
Sodium (mmol/L)	132 (132 – 137)
Potassium (mmol/L)	4.2 (4.2 – 4.9)
Urea (mmol/L)	5 (4.4 – 8.1)
Creatinine (micromol/L)	95 (95 – 135)
Egfr (ml/min/1.73m^2)	70 (49 – 81)
Albumin (g/dL)	33 (31 – 34)
C-reactive protein (mg/L)	229 (218 – 255)
Treatment during follow up period	
Steroids	1 (33.3)
Antibiotics	1 (33.3)
1 year follow up CT imaging (n=3)	
Days from admission to follow up CT	388 (382 – 389)
Days from discharge to follow up CT	371 (354-377)
1 year bronchoalveolar lavage fluid sampling (n=3)	
Days between admission and sampling	410 (402 – 412)
Days between discharge and sampling	392 (374 – 401)
1 year Lung function tests [n=3]	
Days between admission and lung function tests	394 (389 – 409)
Days between discharge and lung function tests [	378 (366-391)
FEV1 (L)	3.3 (2.9-3.9)
% predicted FEV1	104 (97-123)
FVC (L)	3.9 (3.5 – 4.4)
% predicted FVC	101 (91 – 106)
TLC (L)	5.7 (5.6 – 6.7)
% predicted TLC	94 (91 – 95)
TLCO ml/min/mmHg	8.8 (7.3 – 9.9)

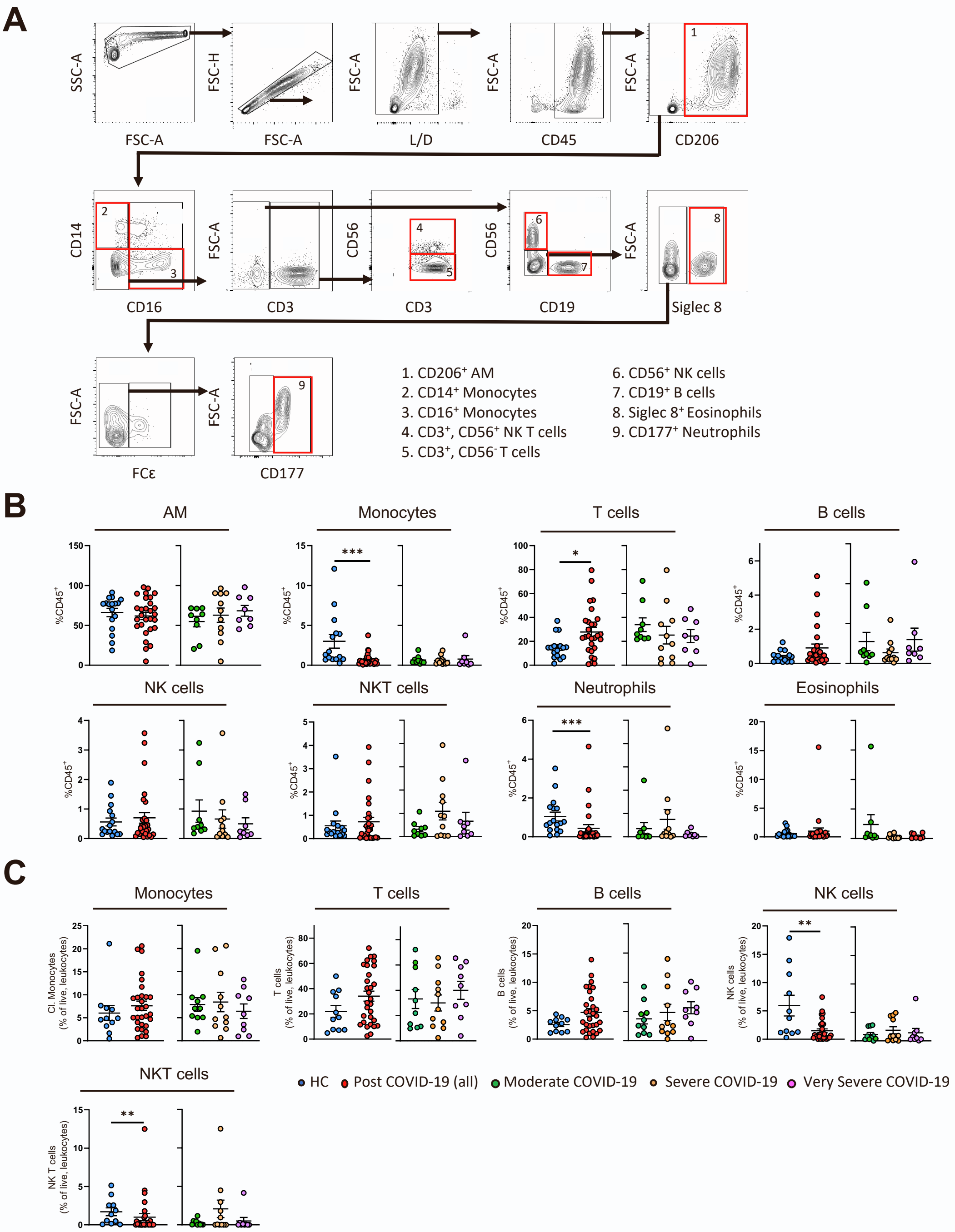

Figure S1: Immune cell proportions in post COVID-19 BAL. Related to Figure 2.
(A) Gating strategy to determine immune cell populations in BAL from healthy controls and pCOVID patients. **(B)** Proportional representation of immune cell populations in BAL from healthy controls and post-COVID19 patients. Healthy controls n = 16, post COVID-19 patients n = 28 (moderate n = 9, severe n = 11, very severe n = 8). **(C)** Proportions of immune populations in peripheral blood from healthy controls and post COVID-19 patients. Healthy controls n = 11, post COVID-19 patients n = 30 (moderate n = 10, severe n = 11, very severe n = 9). (B-C) Post-COVID19 patients were split by severity of acute disease. Data are presented as mean ± SEM. Statistical significance was tested by Mann Whitney U test, Kruskal Wallis test + Dunn's multiple comparison test or Spearman Rank test. *P < **P < 0.01. AM = airway macrophages.

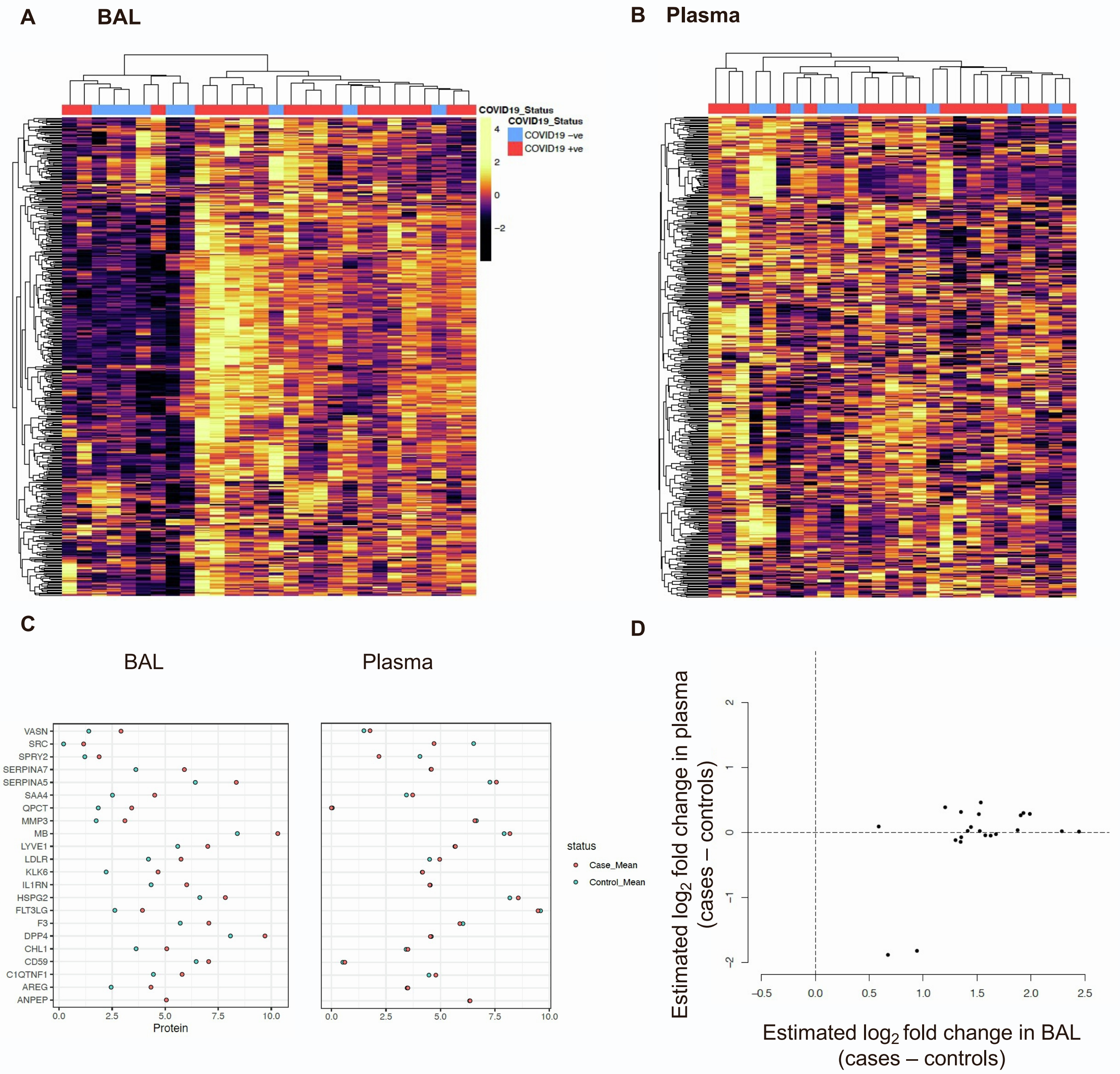

Figure S2. Post-COVID19 effects on the airway proteome are not mirrored in the blood. Related to Figure 3.
(A-B) Hierarchical clustering of samples and proteins using all 435 proteins measured in A) BAL and B) plasma. Z score normalized protein levels are shown. **(C)** Mean NPX values for the 22 proteins that were significantly differentially abundant (5% FDR) in BAL from post-COVID-19 patients versus healthy controls. **(D)** Comparison of effect size estimates in BAL versus plasma for the 22 proteins. Each point represents a protein. The x-axis shows the estimated $\log_2$ fold change in BAL while the y-axis shows the estimated $\log_2$ fold change in plasma.

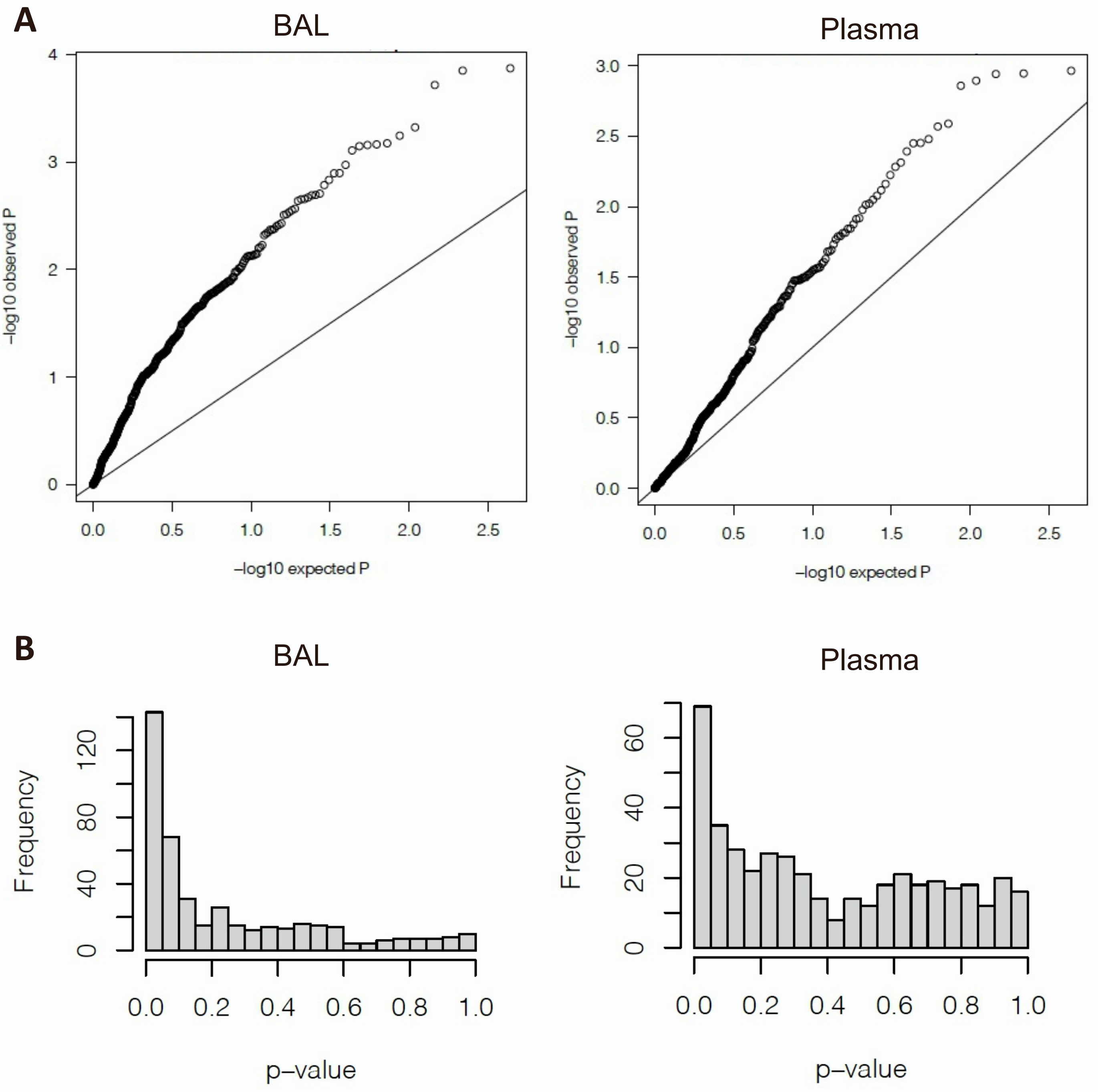

Figure S3. Statistical evidence for proteomic differences between post-COVID19 and healthy controls. Related to Figure 3.
(A) QQ plots showing the expected distribution of –log10 P-values under the null hypothesis of no association between each protein and case/control status compared to the actual –log10 P-values observed in the analysis. The plots shows departure from the diagonal, more marked in BAL. **(B)** Histogram of observed nominal p-values from linear regression of each protein on case/control status. The p-values are not uniformly distributed between 0 and 1. Again this is more marked in BAL.

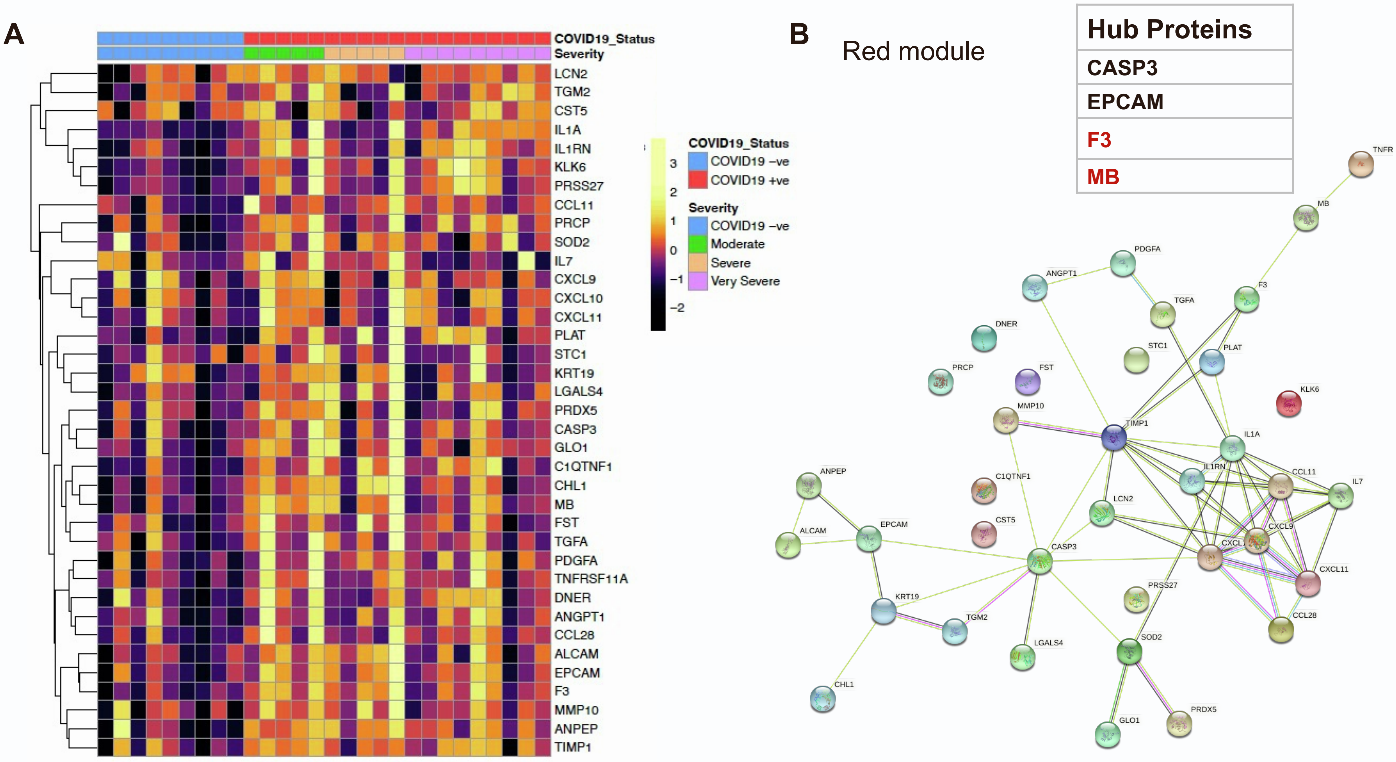

Figure S4: A network of proteins linked to immune cell chemotaxis and cell death is upregulated in the BAL post-COVID19. Related to Figure 3.

WGCNA of BAL proteome of post-COVID19 patients and healthy controls. **(A)** Heatmap displaying Z-score normalised protein abundance for the 37 proteins that form the 'red' protein module. Samples are ordered according to clinical status. Severity refers to peak severity of the acute COVID19 episode. Proteins are ordered by hierarchical clustering. **(B)** Bottom: Network representation of proteins in the red module and their interconnections defined using String-db. An edge in the network represents a relationship between proteins, coloured according to the type of evidence for the connection (see Methods). Top: list of hub proteins within the network (with hub proteins that were also significantly differentially upregulated in post-COVID19 patient BAL highlighted in red).

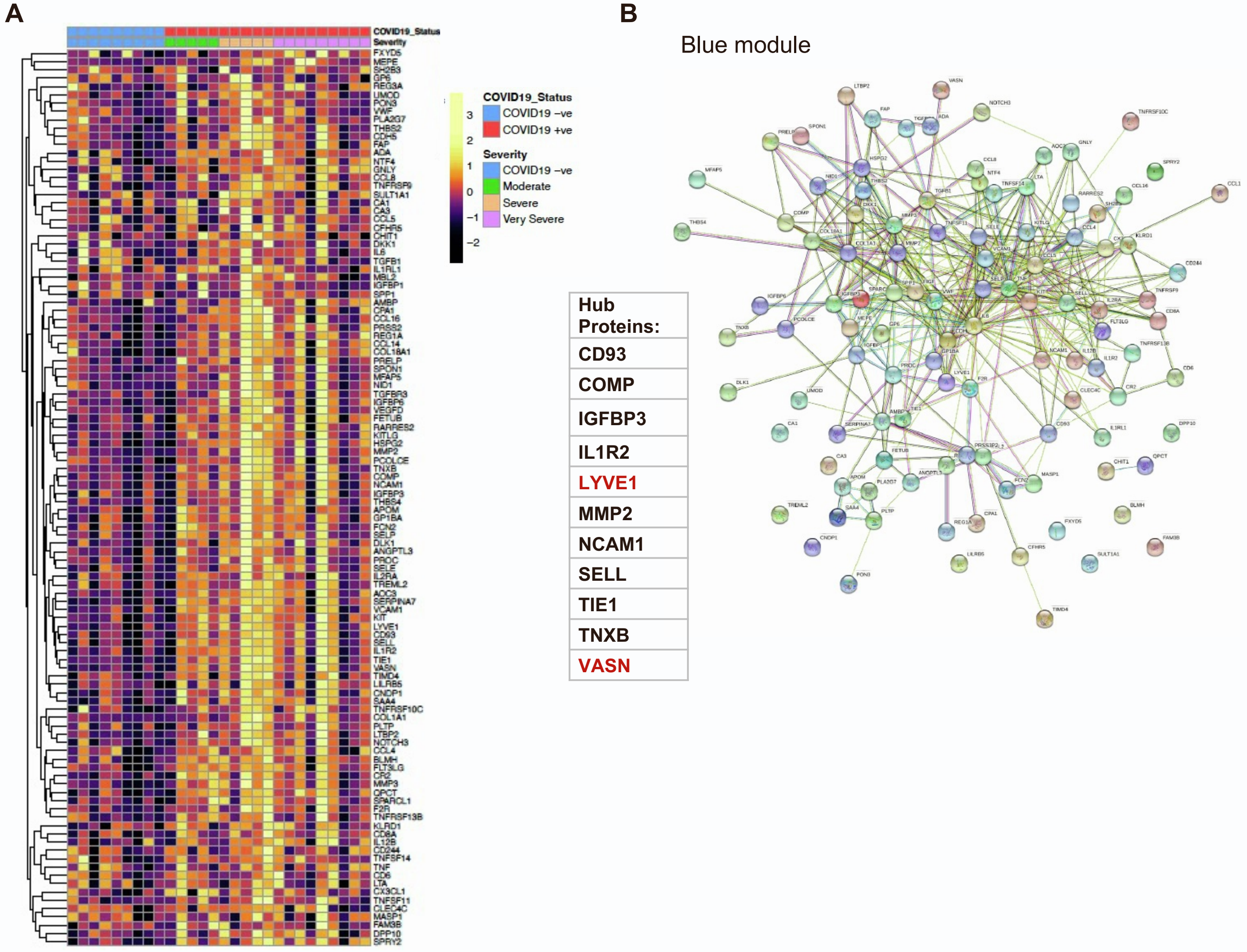

Figure S5. WGCNA identification of the blue module protein network in the post-COVID19 airway. Related to Figure 3.

(A) heatmap displaying Z-score normalised protein abundance for the proteins that form the "blue" eigengene protein module in post-COVID19 and healthy controls in BAL. **(B)** Network representation of proteins in the 'blue' module and their interconnections. An edge in the network represents a relationship between proteins defined using String-db. Predicted hub proteins within the network are stated.

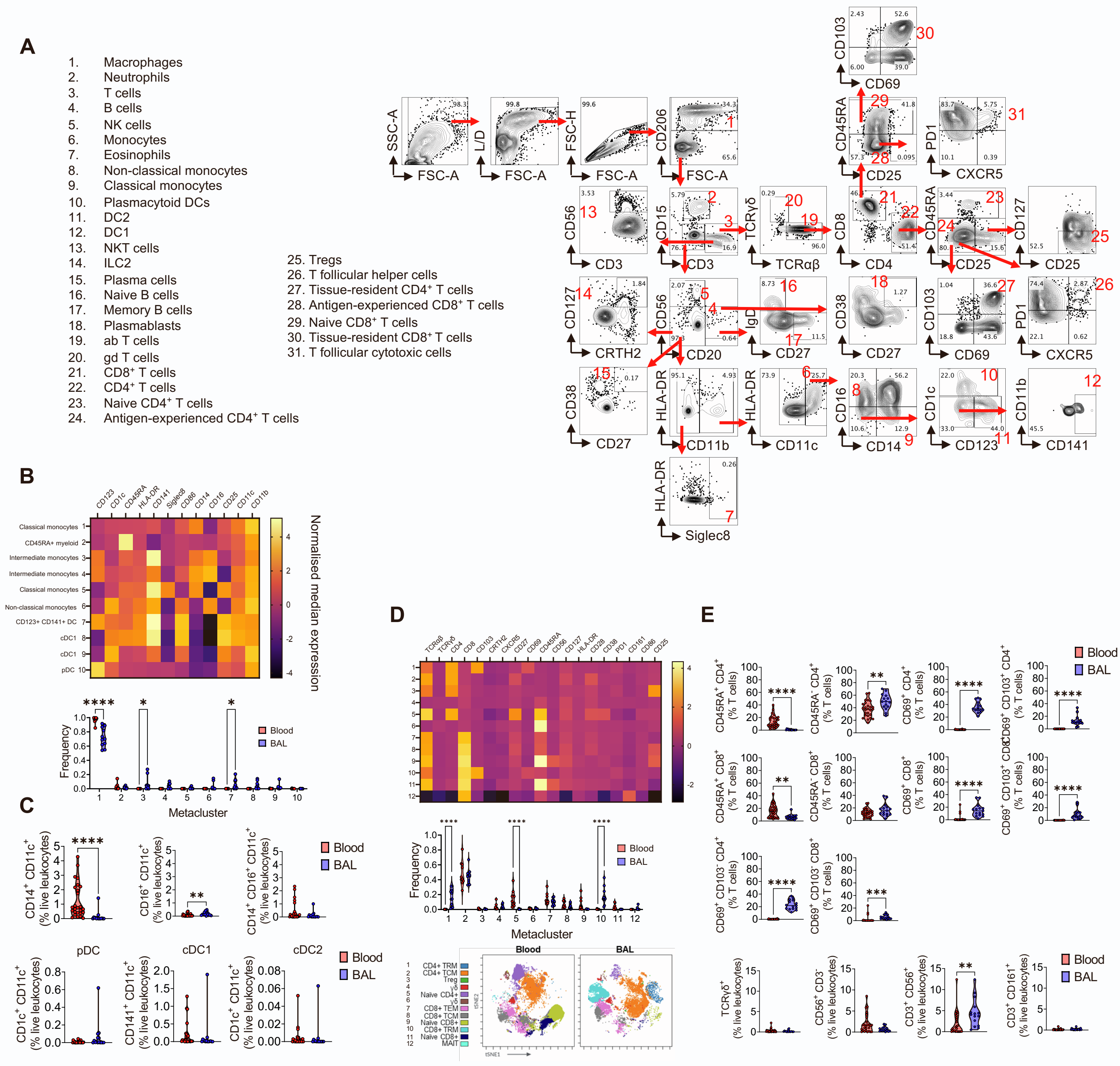

Figure S6. Myeloid and lymphocyte phenotypes in the BAL and blood post COVID-19 fit known subset definitions. Related to Figure 5. (A) Gating strategy for 34-marker spectral deconvolution flow ctyometry panel. Post-COVID BAL cells are shown. **(B)** Heatmap of normalised median expression of myeloid cell modulatory and subset markers by clusters of myeloid cells in the airways identified by FlowSOM analysis. Violin plot showing frequencies of each cluster in BAL and blood. **(C)** Violin plots showing classical, non-classical and intermediate monocyte subsets pDC, cDC1 and cDC2 as proportions of live leukocytes in BAL and blood identified by manual gating. **(D)** Heatmap of normalised median expression of T cell modulatory and subset markers by clusters of T cells in the airways identified by FlowSOM analysis and violin plot showing frequencies of each cluster in BAL and blood. A tSNE projection of clusters identified by FlowSOM analysis in BAL and blood is also provided. **(E)** Violin plots showing CD4⁺ and CD8⁺ T cell subsets as proportions of all T cells and gd, NK, NKT and MAIT cell proportions in BAL and blood. in BAL and blood identified by manual gating. Data depicts n = 15-20 pCOVID patients. Individual data points are shown. Mann-Whitney U-test was carried out. *p < 0.05, ** p < 0.01, *** p<0.001, **** p<0.0001

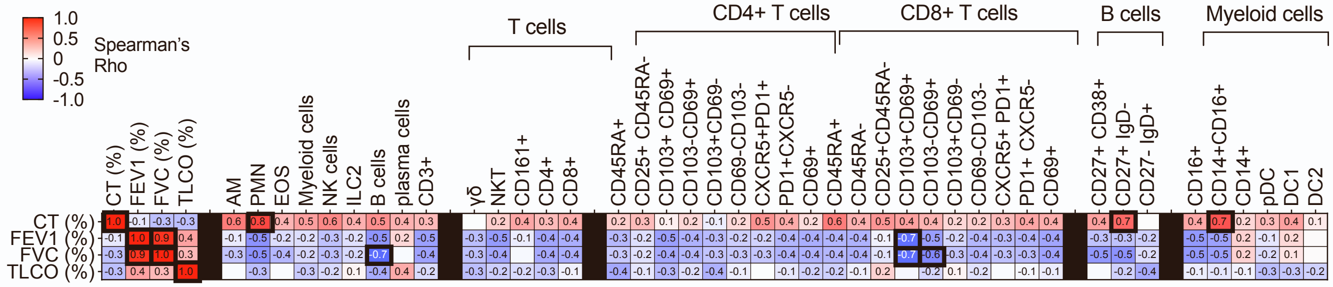

Figure S7. Related to Figure 5. Distinct clinical measurements are associated different immune cell numbers in the post-COVID19 BAL. Spearman's rho of clinical measures of respiratory healthy versus immune cell frequencies in the BAL, as number per ml BAL fluid, were calculated. Correlations $p < 0.05$ after Benjamini-Hochberg adjustment for an FDR of 5% are indicated by boxes.
